# Long-term effects of coronavirus disease 2019 on the cardiovascular system, CV COVID registry: A structured summary of a study protocol

**DOI:** 10.1371/journal.pone.0255263

**Published:** 2021-07-29

**Authors:** Victor Arévalos, Luis Ortega-Paz, Diego Fernandez-Rodríguez, Víctor Alfonso Jiménez-Díaz, Jordi Bañeras, Gianluca Campo, Miguel Rodríguez-Santamarta, Armando Pérez de Prado, Antonio Gómez-Menchero, José Francisco Díaz Fernández, Claudia Scardino, Nieves Gonzalo, Alberto Pernigotti, Fernando Alfonso, Ignacio Jesús Amat-Santos, Antonio Silvestro, Alfonso Ielasi, José María de la Torre, Gabriela Bastidas, Josep Gómez-Lara, Manel Sabaté, Salvatore Brugaletta, on behalf of the CV COVID-19 Registry Investigators

**Affiliations:** 1 Department of Cardiology, Clinic Cardiovascular Institute, Hospital Universitari Clinic, Barcelona, Spain; 2 Department of Cardiology, Hospital Universitari Arnau de Vilanova, Lérida, Spain; 3 Department of Cardiology, Hospital Universitario de Vigo, Vigo, Spain; 4 Department of Cardiology, Hospital Universitari Vall d’Hebron, Barcelona, Spain; 5 Department of Cardiology, Azienda Ospedaliero-Universitaria di Ferrara, Ferrara, Italy; 6 Department of Cardiology, Hospital Universitario de León, León, Spain; 7 Department of Cardiology, Hospital Universitario Juan Ramón Jiménez, Huelva, Spain; 8 Department of Cardiology, Hospital Universitari Joan XXIII, Tarragona, Spain; 9 Department of Cardiology, Hospital Universitario Clínico San Carlos Madrid, Madrid, Spain; 10 Department of Cardiology, Hospital de Tortosa Verge de la Cinta, Tarragona, Spain; 11 Department of Cardiology, Hospital Universitario La Princesa, Madrid, Spain; 12 Department of Cardiology, Hospital Clínico Universitario de Valladolid, Valladolid, Spain; 13 Department of Cardiology, Ospedale Bolognini di Seriate, Bérgamo, Italy; 14 Department of Cardiology, Istituto Clinico Sant’Ambrogio, Milano, Italy; 15 Department of Cardiology, Hospital Marqués de Valdecilla, Santander, Spain; 16 Department of Cardiology, Hospital Universitari Sagrat Cor, Barcelona, Spain; 17 Department of Cardiology, Hospital Universitari de Bellvitge, Barcelona, Spain; Erasmus Medical Centre: Erasmus MC, NETHERLANDS

## Abstract

**Background:**

Patients presenting with the coronavirus-2019 disease (COVID-19) may have a high risk of cardiovascular adverse events, including death from cardiovascular causes. The long-term cardiovascular outcomes of these patients are entirely unknown. We aim to perform a registry of patients who have undergone a diagnostic nasopharyngeal swab for SARS-CoV-2 and to determine their long-term cardiovascular outcomes.

**Study and design:**

This is a multicenter, observational, retrospective registry to be conducted at 17 centers in Spain and Italy (ClinicalTrials.gov number: NCT04359927). Consecutive patients older than 18 years, who underwent a real-time reverse transcriptase-polymerase chain reaction (RT-PCR) for SARS-CoV2 in the participating institutions, will be included since March 2020, to August 2020. Patients will be classified into two groups, according to the results of the RT-PCR: COVID-19 positive or negative. The primary outcome will be cardiovascular mortality at 1 year. The secondary outcomes will be acute myocardial infarction, stroke, heart failure hospitalization, pulmonary embolism, and serious cardiac arrhythmias, at 1 year. Outcomes will be compared between the two groups. Events will be adjudicated by an independent clinical event committee.

**Conclusion:**

The results of this registry will contribute to a better understanding of the long-term cardiovascular implications of the COVID19.

## Introduction

Since Coronavirus disease 2019 (COVID-19) was first reported in China in late December 2019, it has spread rapidly worldwide, and it has become a pandemic affecting more than 200 countries. The exponential increase in the number of COVID-19 patients has overwhelmed health-care systems in many countries across the world, with an unprecedented effect not only on public health, but also on social and economic activities.

COVID-19 is caused by the severe acute respiratory syndrome coronavirus-2 (SARS-CoV-2). Currently, it is known that SARS-CoV-2 can produce a multi-system affectation, including cardiovascular system [1]. This virus enters the cells by binding of the viral spike (S) protein to angiotensin-converting enzyme 2 (ACE2) on the surface of the host cell. The ACE2 is highly expressed in pulmonary tissues, but also in adult human hearts and endothelial cells, indicating an intrinsic susceptibility of these organs to a direct invasion of SARS-CoV-2. Furthermore, SARS-CoV-2 probably produces a downregulation in the ACE2 activity, reducing the conversion of angiotensin II (Ang II) to Ang-(1–7). This may increase Ang II activity, which continues stimulating renin-angiotensin-aldosterone system (RAAS) with deleterious effects on heart and blood vessels [2–4].

An exacerbated inflammatory response with cytokine storm mediated through pathologic T cells and monocytes leading to myocarditis, is another possible mechanism postulated to explain cardiac injury due to COVID-19 [5, 6]. The pro-inflammatory environment and endothelial dysfunction can also trigger the development of coagulopathy. Hypercoagulability leads to coronary macro and microvascular thrombosis, and high incidence of thromboembolic events in venous territories [7–9].

We currently know that myocardial injury is related with worse in-hospital prognosis in COVID-19 patients [10–12]. A study of 26 patients recovered from COVID-19 evaluated with cardiac resonance showed myocardial edema, fibrosis, and impaired right ventricle function [13]. Available data suggests a probable implication on prognosis, but long-term implications of COVID-19 on cardiovascular events are completely unknown.

The Cardiovascular COVID-19 (CV COVID-19) registry will aim to a better understanding of the long-term cardiovascular implications in patients who had a SARS-CoV-2 infection.

## Methods

### Study design

It is a multicenter, observational, retrospective registry to be conducted at 17 centers in Spain and Italy. This study is an investigator-initiated registry, and the promoter is the Fundació Clínic per a la Recerca Biomédica. **Table 1** shows the list of enrolling centers and their principal investigators. The detailed list of the study staff is in the **S1 File**.

**Table 1:** Centers and investigators.

Centers	Investigators
Hospital Universitari Clínic de Barcelona	Dr. Luis Ortega-Paz
Hospital Universitari Sagrat Cor	Dr. Gabriela Bastidas
Hospital Universitari de Bellvitge	Dr. Josep Gómez-Lara
Hospital Marqués de Valdecilla	Dr. José María de la Torre
Hospital Universitario Juan Ramón Jiménez	Dr. Antonio Gómez-Menchero, Dr. José Francisco Díaz Fernández
Hospital Universitario La Princesa	Dr. Fernando Alfonso
Hospital Universitari Vall d’Hebron	Dr. Jordi Bañeras Rius
Hospital Clínico Universitario de Valladolid	Dr. Ignacio Jesús Amat-Santos
Hospital Universitario de León	Dr. Miguel Rodríguez-Santamarta
Hospital Universitario de Vigo	Dr. Victor Alfonso Jimenez-Díaz
Hospital Universitario Clínico San Carlos	Dr. Nieves Gonzalo
Istituto Clinico Sant’Ambrogio	Dr. Alfonso Ielasi
Azienda Ospedaliero-Universitaria di Ferrara	Dr. Gianluca Campo
Hospital de Tortosa Verge de la Cinta	Dr. Alberto Pernigotti
Ospedale Bolognini di Seriate	Dr. Antonio Silvestro
Hospital Universitari Arnau de Vilanova	Dr. Diego Fernández-Rodríguez
Hospital Universitari Joan XXIII	Dr. Claudia Scardino

The Registry does not test clinical interventions, and individual patient care is entirely at the discretion of treating clinicians.

This study adhered to the principles outlined in the Declaration of Helsinki. Approval was given by the “Comité de Ética de la Investigación con medicamentos del Hospital Clínic de Barcelona” (Ethics Committee for Drug Research of the Hospital Clínic of Barcelona) with the registry HCB/2020/0457, on April 16, 2020. Given the registry’s anonymous characteristics and the health alarm situation generated by the COVID-19 pandemic, written informed consent was waived.

The study is registered at ClinicalTrials.gov (NCT number: NCT04359927).

### Patient selection

Inclusion criteria are:

1. At least 18 years of age.
2. Patient who underwent a nasopharyngeal swab for real-time reverse transcriptase-polymerase chain reaction (RT-PCR) for SARS-CoV2 between March 2020 and August 2020.

Exclusion criteria are:

Those with terminal diseases and a life expectancy <1 year before the diagnosis will be excluded.

Patients will be classified into two groups: patients with confirmed COVID-19 (positive RT-PCRT for SARS-CoV-2) and patients without COVID-19 (negative RT-PCR for SARS-CoV2, and absence of absence of suspicious symptoms).

### Data capture

Study data will be collected and managed using REDCap electronic data capture tools hosted at Hospital Clínic of Barcelona (redcap.clinic.cat). REDCap (Research Electronic Data Capture) is a secure, web-based software platform designed to support data capture for research studies, providing 1) an intuitive interface for validated data capture; 2) audit trails for tracking data manipulation and export procedures; 3) automated export procedures for seamless data downloads to common statistical packages; and 4) procedures for data integration and interoperability with external sources.

An anonymized and predefined electronic Case Report Form (eCRF) developed by the investigators will be filled by each participating center. The selected variables are oriented to the cardiovascular risk factors, conditions, medications, and outcomes (**Table 2**). Moreover, based on the current scientific literature, specific COVID-19 variable and treatment will be also collected. Charlson Comorbidity Index (CCI) will be used to assess patient’s comorbidities. CCI is a validated index for predicting life expectancy at ten years, depending on the age at which it is evaluated, and on the subject’s comorbidities [14]. The detailed eCRF format can be seen in the **S2 File**.

**Table 2:** Data to be obtained in the timeline of the study.

Baseline characteristics	Acute phase	Long-term (until 1 year)
Demographic data • Age • Sex Risk factors and baseline comorbidities • Hypertension • Diabetes mellitus • Hypercholesterolemia • Smoking status • Kidney chronic disease • Previous ACS • Previous Stroke • Heart failure • Atrial fibrillation • Previous PE and DVT • Cancer • Organ transplant • Immunosupresion • Frailty Charlson Comorbidity Index Baseline medication • Anti-hypertensive drugs and cardiovascular medication • Chronic anticoagulation • Oral hypoglycemic agents • Insulin • Proton pump inhibitors • NSAIDs	Symptoms Hospitalization • Length of stay Drugs: • Anti-hypertensive drugs and cardiovascular medication. • Anticoagulation • Antivirals • Immunosuppressive drugs • Vasopressors • ICU admission • Invasive mechanical ventilation Biomarkers • Hemoglobin* • Lymphocytes* • Platelets* • Creatinine** • HscTnI** • NT-proBNP** • D-dimer** • Fibrinogen* • Prothrombin time** In-hospital events: • All-cause death • Cardiovascular death • ACS • Stroke • DVT and PE • Cardiac arrhytmias • Bleeding • Red blood cell transfusion Discharge medication	New SARS-CoV-2 infection Vaccination against SARS-CoV-2 Outcomes • All cause death • Cardiovascular death • ACS • Stroke • Heart failure hospitalization • PE • Cardiac arrhytmias

ACS, acute coronary syndrome; DVT, deep venous thrombosis; Hs-cTnI, high sensitivity cardiac troponin I; NSAIDs, non-steroidal anti-inflammatory drugs; PE, pulmonary embolism.

*Lowest values during hospitalization.

*Highest values during hospitalization.

All the data will be obtained from electronic records (medical history). If deemed necessary, the investigator may check the patient vital status in the social national security database.

**Fig 1:**
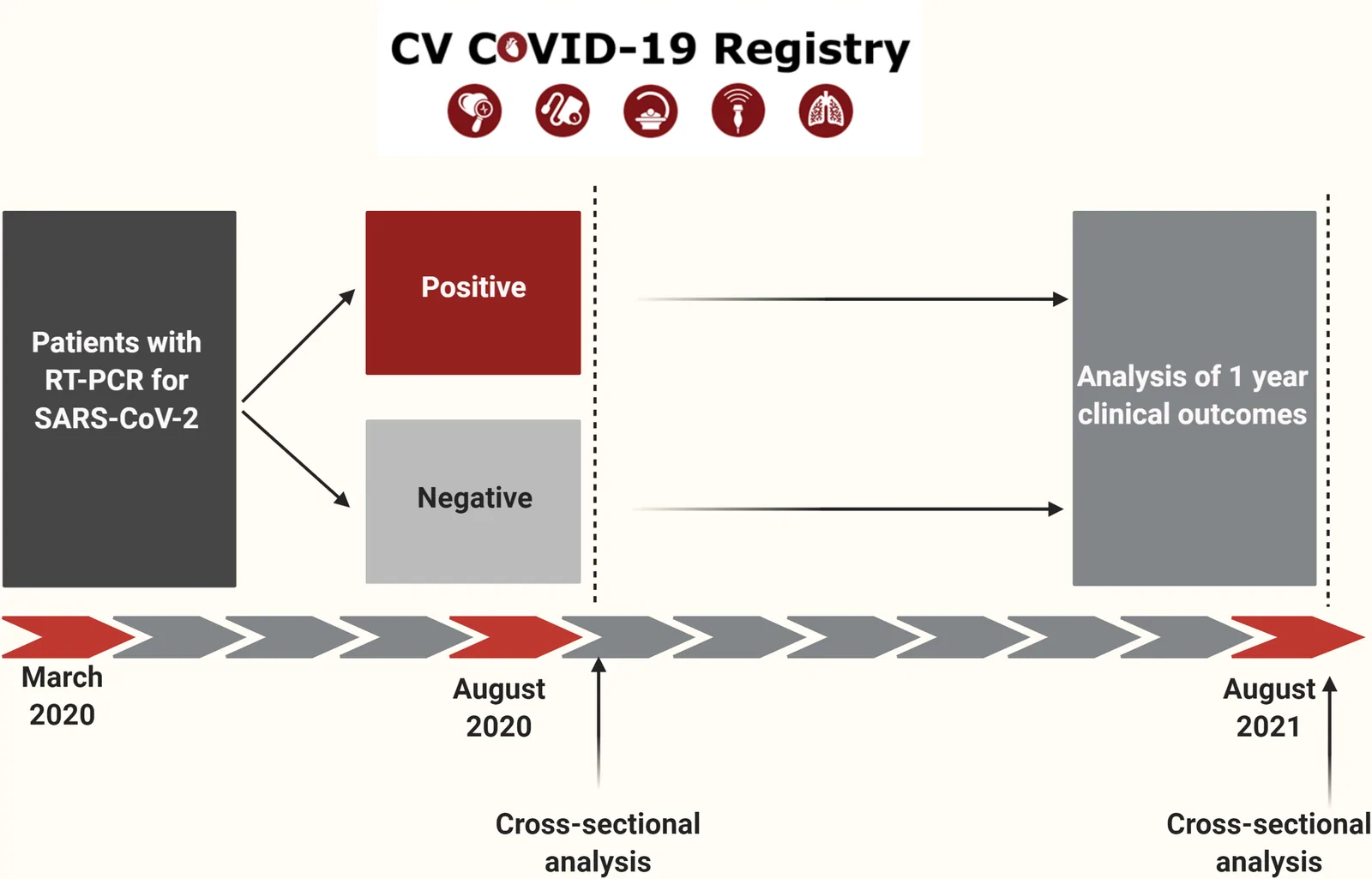
Timeline of the study. Inclusion of the patients will be since March to August 2020. Analysis of the clinical outcomes will be performed in August 2021.

Clinical follow-up will be performed at 1-year. The flowchart and study timeline are detailed in **Fig 1**.

### Monitoring

Independent study monitors (Effice, Madrid, Spain) verified the adequacy of the follow-up and events reported, conducting auditing in a random sample of 10% of all patients included. All events were adjudicated and classified by an independent event adjudication committee (**S1 Table**) blinded to the treatment groups by reviewing source documents (including angiograms) provided by each center (Barcicore Lab, Barcelona, Spain).

### Outcomes and definitions

The primary outcome will be:

- Cardiovascular mortality at 1-year follow-up, defined according to the Academic Research Consortium-2 [15].

The secondary outcomes will be:

- Myocardial infarction: defined according to the Academic Research Consortium-2 [15].
- Stroke: defined according to the Academic Research Consortium-2 [15].
- Heart failure hospitalization must be documented in the diagnosis of the hospitalization discharge letter.
- Pulmonary embolism must be documented with a computed tomography.
- Cardiac arrhythmias must be documented in the diagnosis of the hospitalization discharge letter. Serious cardiac arrhythmias were defined as: bradycardia requiring intravenous medication or pacemaker, supraventricular tachycardia requiring intravenous medication or cardioversion, or ventricular tachycardia requiring intravenous medication or cardioversion.
- Major bleeding was defined as a type 3 of the Bleeding Academic Research Consortium (BARC) or higher [16].

All the events will be independently adjudicated by a Clinical Events Committee. The Clinical Event Committee (CEC) consists of cardiologists not participating in the trial. The CEC members will be blinded to PCR COVID of the patient.

### Statistical analysis plan

We did not estimate a precise sample size, due to lack of literature reports and we aim to get the maximum numbers of patients possible.

Continuous variables will be presented as mean ± standard deviation. Categorical variables will be reported as absolute number and percentage. Differences in proportions will be tested with Chi-square test or Fishers exact test and differences in continuous variables will be tested with a Student’s t-test. Kaplan-Meier method will be used to derive the event rates at follow-up and to plot time-to-event curves. Patients not eligible for 1-year follow-up will be considered at risk until the date of last contact, at which point they will be censored.

To determine the predictors of cardiovascular death, a Cox proportional hazards model will be used together with the Wald test to compare the results between the groups (patients with positive vs. negative RT-PCR for SARS-CoV-2).

All p-values will be two-sided and a value <0.05 will be considered statistically significant. All data will be processed using the Statistical Package for Social Sciences, version 22 (SPSS Inc., Chicago, IL, USA).

## Discussion

In this multicenter, observational, retrospective registry, we aim of investigate the long-term cardiovascular implications of COVID-19. Currently is known that the presence of myocardial injury, vascular dysfunction and thrombosis in patients with COVID-19 have an important role in the short-term prognosis in these patients [9]. Previous experience with the severe acute respiratory syndrome coronavirus-1 (SARS-CoV-1) emerged in 2002, suggests that both the underlying disease and its treatment could be associated with a worse cardiovascular prognosis. In a study of 25 survivors of SARS-CoV-1, at 12 years of follow-up, altered lipid metabolism was found [17]. Similarly, viral diseases such as influenza A are associated with increased cardiovascular mortality after infection [18].

The focus of this registry will be on the cardiovascular outcomes, providing data not only about cardiac ischemic events but also arrhythmias, cerebrovascular events and heart failure. It should be highlighted that our findings will be applicable to any COVID19 patient, regardless of the severity of the disease, as we will be including a consecutive population of patients with COVID-19 infection. Confirmed cases will be evaluated and compared with those patients who did not have COVID-19 infection. This approach will offer a greater security at the moment of assessing study’s findings. This control group will help to minimize bias that would arise if only COVID-19 patients were independently analyzed. Cox regression will help to distinguish the independent predictors for mortality among the patients of the study. However, considering that a proportion of the SARS-CoV-2 infections can be asymptomatic, there can be a degree of bias if the control patients (non-COVID-19 group) become infected but exhibit an asymptomatic disease during follow-up. Nevertheless, potential patients fulfilling these characteristics can represent a minimal part of the sample size.

It is important to mention as strength of the study, that the events will be adjudicated by an independent committee blinded to the patients’ treatment allocation and trial results. This will guarantee a better evaluation of the study events.

## Conclusion

The results of the CV COVID-19 registry will contribute to a better understanding of the short and long-term implications of this disease. It will provide information about cardiovascular mortality and cardiac events at one year of follow-up. Also, it will identify characteristics associated with higher incidence of complications, defining a profile of COVID-19 patients with worse prognosis.

## Supporting information

S1 Table List of committees and members. (DOCX)

S1 File List of all investigators of the involved centers. (DOCX)

S2 File Electronic case report form. (PDF)

S1 Data (PDF)
