# Supplementary material for: Long-term effects of coronavirus disease 2019 on the cardiovascular system, CV COVID registry: A structured summary of a study protocol
Source: PLoS One. 2021 Jul 29;16(7):e0255263. doi: 10.1371/journal.pone.0255263 (PMC8320971; doi:10.1371/journal.pone.0255263)
Supplement: S1 Table — (DOCX) [file pone.0255263.s001.docx]

| **Committee** | **List of Members** |
| --- | --- |
| Steering Committee | Dr. Luis Ortega-Paz; Hospital Clínic; Barcelona; Spain  Dr. Salvatore Brugaletta; Hospital Clínic; Barcelona; Spain  Dr. Manel Sabaté; Hospital Clínic; Barcelona; Spain  Dr. José María de la Torre; Hospital Marqués de Valdecilla |
| Clinical Event Committee | Barcicore Cardiac Imaging Corelab, Barcelona, Spain  Dr. Josep Gómez-Lara |
| CRO and monitoring | Effice, Madrid, Spain |
| Statistics | Effice, SL, Madrid, Spain  Mr. José Montes, study statistician |
