# Supplementary material for: Long-term effects of coronavirus disease 2019 on the cardiovascular system, CV COVID registry: A structured summary of a study protocol
Source: PLoS One. 2021 Jul 29;16(7):e0255263. doi: 10.1371/journal.pone.0255263 (PMC8320971; doi:10.1371/journal.pone.0255263)
Supplement: S1 File — (DOCX) [file pone.0255263.s002.docx]

**Long-term Effects of Coronavirus Disease 2019 on the Cardiovascular System: CV COVID-19 Registry (NCT04359927)**

**Investigators:**

**Spain:** Luis Ortega-Paz, Víctor Arévalos, Juan José Rodríguez-Arias, Manel Sabaté, Salvatore Brugaletta, Diego Fernández, Pablo Pastor Pueyo, Antonio Esmo, Moya Halley, Ubaldo Hernández, Enrique Enzo, Pablo Juan, Víctor Jiménez Díaz, Montse Bach, Pablo Jordan, Jordi Bañeras Rius, Samuel Del Castillo García, Carlos Minguito Carazo, Julio Echarte Morales, Miguel Rodríguez Santamarta, Antonio Gómez, José Francisco Díaz Fernández, Josefa García, Zaira Gómez, Teresa Romero, Nieves Gonzalo, Fernando Alfonso, Ignacio Amat Santos, Gabriela Bastidas Mora, Josep Gómez-Lara, Manuel Lozano, Miguel Molina, José María de la Torre Hernández, Alberto Pernigotti, Claudia Scardino. **Italy**: Ottavio Zucchetti, Gianluca Campo, Alfonso Ielasi, Antonio Silvestro.
