# Supplementary material for: Long-term effects of coronavirus disease 2019 on the cardiovascular system, CV COVID registry: A structured summary of a study protocol
Source: PLoS One. 2021 Jul 29;16(7):e0255263. doi: 10.1371/journal.pone.0255263 (PMC8320971; doi:10.1371/journal.pone.0255263)
Supplement: S2 File — (PDF) [file pone.0255263.s003.pdf]

CV COVID-19 Registry PID 162

Project Home | Project Setup | Online Designer | Data Dictionary | Codebook

Data Dictionary Codebook

27.06.2021 0

Collapse all instrumer

#	Variable / Field Name	Field Label<br>*Field Note*	Field Attributes (Field Type, Validation, Choices, Calculations, etc.)
Instrument: **Center & Patient ID** (center_patient_id)			Collaps
1	record_id	Record ID	text
2	nhc	Number of clinical history<br>*Of the hospitalization center*	text, Required, Identifier
3	dob	Date of Birth	text (date_dmy, Max: 2020-02-24), Required
4	sex	Gender	radio, Required<br>1 Men<br>2 Women
5	coordinating_center	Did the patient was enrolled in the coordinating center?	yesno, Required<br>1 Yes<br>0 No
6	postal_code	Postal code (código postal)<br>*ONLY FOR BARCELONA CENTERS (SOLO PARA CENTROS DE BARCELONA)*	text (number)
7	center_patient_id_complete	Section Header: *Form Status*<br>Complete?	dropdown<br>0 Incomplete<br>1 Unverified<br>2 Complete
Instrument: **Baseline** (baseline)			Collaps
8	base_height_cm	Section Header: *BASELINE - CLINICAL CHARACTERISTICS (If any characteristic is unknown, use the NO option)*<br>Height (cm)<br>*If unknown use 999.*	text (number, Min: 50, Max: 999), Required
9	base_weight_kg	Weight (Kg)<br>*If unknown use 999.*	text (number, Min: 40, Max: 999), Required
10	base_smoking	Previous smoking status	radio, Required<br>0 Never<br>1 Current<br>2 Former
11	base_diabetes	Previous Diabetes mellitus	yesno, Required<br>1 Yes<br>0 No
12	base_hypertension	Previous Hypertension	yesno, Required<br>1 Yes<br>0 No
13	base_hypercholesterolemia	Previous Hypercholesterolemia	yesno, Required<br>1 Yes<br>0 No
14	base_ckd	Previous Chronic Kidney Disease<br>*For eGFR calculation: https://www.mdcalc.com/ckd-epi-equations-glomerular-filtration-rate-gfr*	radio, Required<br>1 No<br>2 Yes - eGFR < 60 ml/min/1.73m2<br>3 Dialysis

15	base_atrial_fib	Previous Atrial fibrillation or Atrial flutter	yesno, Required<br>1 Yes<br>0 No
16	base_family_hist_cad	Family History of Premature Coronary Artery Disease<br>*< 55 years in first-degree male relatives and < 65 years in female relatives.*	yesno, Required<br>1 Yes<br>0 No
17	base_stroke_tia	Previous Stroke or transient ischemic attack	yesno, Required<br>1 Yes<br>0 No
18	base_mi	Previous Myocardial infarction	yesno, Required<br>1 Yes<br>0 No
19	base_pci	Previous Percutaneous coronary intervention	yesno, Required<br>1 Yes<br>0 No
20	base_cabg	Previous Coronary artery bypass grafting (CABG)	yesno, Required<br>1 Yes<br>0 No
21	base_pvd	Previous Peripheral Vascular Disease	yesno, Required<br>1 Yes<br>0 No
22	base_copd_asthma	Previous Chronic obstructive pulmonary disease (COPD) or Asthma	yesno, Required<br>1 Yes<br>0 No
23	base_pneumonia	Previous history of pneumonia<br>*Any type*	yesno, Required<br>1 Yes<br>0 No
24	baseline_hf	Previous Heart Failure	yesno, Required<br>1 Yes<br>0 No
25	base_hf_nhya<br>Show the field ONLY if:<br>[baseline_hf] = '1'	If the patient has previous heart failure, specify the New York Heart Association (NYHA) classification.<br>*https://es.wikipedia.org/wiki/Clasificación_funcional_New_York_Heart_Association*	radio, Required<br>1 I<br>2 II<br>3 III<br>4 IV
26	base_pul_hyper	Pulmonary hypertension<br>*Define by clinical history or PA systolic pressure >30 mmHg by echocardiogram.*	yesno, Required<br>1 Yes<br>0 No
27	base_vte_pe	Previous Venous Thromboembolism/Pulmonary Embolism	yesno, Required<br>1 Yes<br>0 No
28	base_bleeding	Previous Major bleeding<br>*Any history of bleeding requiring medical attention (i.e. gastrointestinal bleeding, intracranial bleeding, or hemorrhagic stroke, etc.)*	yesno, Required<br>1 Yes<br>0 No
29	base_dementia	Any dementia or cognitive impairment?	radio, Required<br>1 No<br>2 Any cognitive impairment<br>3 Any dementia
30	base_cancer	Previous Cancer	radio, Required

			0 No<br>1 Previous<br>2 Active no treatment<br>3 Active under treatment
31	base_cancer_type<br>Show the field ONLY if:<br>[base_cancer] = '1' or [base_cancer] = '2' or [base_cancer] = '3'	If yes, specify the cancer type	text, Required
32	base_transplantation	The patient has any type of organ transplant?	yesno, Required<br>1 Yes<br>0 No
33	base_transplantation_type<br>Show the field ONLY if:<br>[base_transplantation] = '1'	Which type of organ transplant?	dropdown, Required<br>1 Renal<br>2 Liver<br>3 Heart<br>4 Bone narrow<br>5 Lung<br>6 Pancreatic<br>7 Intestinal<br>8 Renal and pancreatic<br>9 Lung and Heart<br>10 Liver and Heart<br>11 Renal and liver<br>12 Liver, heart and pancreas
34	other_disease_base	If any other relevant disease (i.e. immunosuppression)<br>*Use a comma to separate the diagnoses. Example: lupus, gastrinoma.*	notes
35	base_aspirin	Section Header: *BASELINE - TREATMENT*<br>Aspirin	yesno, Required<br>1 Yes<br>0 No
36	base_p2y12	P2Y12 inhibitor<br>*Example: clopidogrel, prasugrel, Ticagrelor*	yesno, Required<br>1 Yes<br>0 No
37	base_p2y12_type<br>Show the field ONLY if:<br>[base_p2y12] ='1'	P2Y12 inhibitor	radio, Required<br>1 Clopidogrel<br>2 Prasugrel<br>3 Ticagrelor
38	base_ace_inh	ACE inhibitor<br>*Angiotensin-converting-enzyme inhibitors (ACE inhibitors)*	yesno, Required<br>1 Yes<br>0 No
39	base_ace_type<br>Show the field ONLY if:<br>[base_ace_inh] = '1'	ACE inhibitor type	radio, Required<br>1 Lisinopril<br>2 Enalapril<br>3 Perindopril<br>4 Other
40	base_ace_other<br>Show the field ONLY if:<br>[base_ace_type] = '4'	If other ACE inhibitor, specify which	text, Required
41	base_ace_dose<br>Show the field ONLY if:<br>[base_ace_inh] = '1'	ACE inhibitor TOTAL daily dose (mg)<br>*If unknown use 999.*	text (number, Min: 1, Max: 999)

42	base_arbs	ARBs<br>*Angiotensin II receptor blockers*	yesno, Required<br>1 Yes<br>0 No
43	base_arbs_type<br>Show the field ONLY if:<br>[base_arbs] = '1'	ARBs type	radio, Required<br>1 Losartan<br>2 Candesartan<br>3 Irbesartan<br>4 Telmisartan<br>5 Valsartan<br>6 Olmesartan<br>7 Other
44	base_arbs_other<br>Show the field ONLY if:<br>[base_arbs_type] = '7'	If other ARBs, specify which	text
45	base_abrs_dose<br>Show the field ONLY if:<br>[base_arbs] = '1'	ARBs TOTAL daily dose (mg)<br>*If unknown use 999.*	text (number, Min: 0, Max: 999), Required
46	base_arni	Angiotensin receptor neprilysin inhibitor (ARNI)<br>*Sacubitril/valsartan (Entresto)*	yesno, Required<br>1 Yes<br>0 No
47	base_arni_dose<br>Show the field ONLY if:<br>[base_arni] = '1'	Angiotensin receptor neprilysin inhibitor (ARNI) TOTAL daily dose (mg)<br>*Sacubitril/valsartan (Entresto). If unknown use 999.*	text (number, Min: 0, Max: 999)
48	base_statins	Statins	yesno, Required<br>1 Yes<br>0 No
49	base_statin_type<br>Show the field ONLY if:<br>[base_statins] = '1'	Statin type	radio, Required<br>1 Atorvastatin<br>2 Rosuvastatin<br>3 Simvastatin<br>4 Other
50	base_statin_other<br>Show the field ONLY if:<br>[base_statin_type] = '4'	If other statin, specify which	text, Required
51	base_statin_dose<br>Show the field ONLY if:<br>[base_statins] = '1'	Statin TOTAL daily dose (mg)<br>*If unknown use 999.*	text (number, Min: 0, Max: 999)
52	base_beta_block	Beta Blocker	yesno, Required<br>1 Yes<br>0 No
53	base_beta_block_type<br>Show the field ONLY if:<br>[base_beta_block] = '1'	Beta Blocker type	radio, Required<br>1 Bisoprolol<br>2 Atenolol<br>3 Metoprolol<br>4 Carvedilol<br>5 Other
54	base_beta_block_other<br>Show the field ONLY if:<br>[base_beta_block_type] = '5'	If other Beta blocker, specify which	text, Required
55	base_beta_block_dose<br>Show the field ONLY if:	Beta Blocker TOTAL daily dose (mg)<br>*If unknown use 999.*	text (number, Min: 0, Max: 999)

	[base_beta_block] = '1'		
56	base_ccb	Calcium channel blocker	yesno, Required<br>1 Yes<br>0 No
57	base_ccb_type<br>Show the field ONLY if:<br>[base_ccb] = '1'	Calcium channel blockers type	radio, Required<br>1 Amlodipine<br>2 Diltiazem<br>3 Nicardipine<br>4 Nifedipine<br>5 Verapamil<br>6 Other
58	base_ccb_other<br>Show the field ONLY if:<br>[base_ccb_type] = '6'	If other calcium channel blocker, specify which	text, Required
59	base_ccb_dose<br>Show the field ONLY if:<br>[base_ccb] = '1'	Calcium channel blocker TOTAL daily dose (mg)<br>*If unknown use 999.*	text (number, Min: 0, Max: 999), Required
60	base_loop_diuretics	Loop diuretic<br>*Example: Furosemide, Torsemide, etc.*	yesno, Required<br>1 Yes<br>0 No
61	base_loop_diuretic_dose<br>Show the field ONLY if:<br>[base_loop_diuretics] = '1'	Loop diuretic TOTAL daily dose (mg)<br>*If unknown use 999.*	text (number, Min: 0, Max: 999), Required
62	base_mras	Mineralocorticoid receptor antagonists (MRAs)<br>*Spironolactone or eplerenone*	yesno, Required<br>1 Yes<br>0 No
63	base_mras_type<br>Show the field ONLY if:<br>[base_mras] ='1'	Mineralocorticoid receptor antagonists type	radio, Required<br>1 Spironolactone<br>2 Eplerenone
64	base_mras_dose<br>Show the field ONLY if:<br>[base_mras] ='1'	Mineralocorticoid receptor antagonists (MRAs) TOTAL daily dose (mg)<br>*If unknown use 999.*	text (number, Min: 0, Max: 999), Required
65	base_ppis	Proton-pump inhibitors	yesno, Required<br>1 Yes<br>0 No
66	base_oral_hypo_glc	Oral hypoglycemic agents	yesno, Required<br>1 Yes<br>0 No
67	base_insulin	Insulin	yesno, Required<br>1 Yes<br>0 No
68	base_anticoagulant	Anticoagulant treatment<br>*Example: sintrom, ACODs, enoxaparin, etc.*	yesno, Required<br>1 Yes<br>0 No
69	base_anticoagulant_tx<br>Show the field ONLY if:<br>[base_anticoagulant] = '1'	Which anticoagulant treatment<br>**Example: Rivaroxaban, dabigatran, apixaban, or edoxaban.*	radio, Required<br>1 Vitamin K antagonists (acenocoumarol)<br>2 Directly acting oral anticoagulants*<br>3 Low-molecular-weight heparin (enoxaparin, etc
70	base_noac_type<br>Show the field ONLY if:<br>[base_anticoagulant_tx] = '2'	If Directly acting oral anticoagulants was used which one:	radio, Required<br>1 Rivaroxaban (Xarelto)

	[illegible]		2 Dabigatran (Pradaxa)<br>3 Apixaban (Eliquis)<br>4 Edoxaban (Lixiana)
71	base_noac_dose<br>Show the field ONLY if:<br>[base_anticoagulant_tx] = '2'	Specify the Directly acting oral anticoagulants TOTAL Daily dose (mg)<br>*If unknown use 999.*	text (number, Min: 0, Max: 999), Required
72	base_nsaids	Nonsteroidal anti-inflammatory drugs (NSAIDs)	radio, Required<br>0 No<br>1 Yes - Chronic use<br>2 Yes - Only recent use
73	base_nsaids_type<br>Show the field ONLY if:<br>[base_nsaids] = '1' or [base_nsaids] = '2'	NSAIDs type	radio<br>1 Ibuprofen<br>2 Diclofenac<br>3 Naproxen<br>4 Dexketoprofen or ketoprofen<br>5 Celecoxib<br>6 Other
74	base_nsaids_other<br>Show the field ONLY if:<br>[base_nsaids_type] = '6'	If other Nonsteroidal anti-inflammatory drugs (NSAIDs), specify which	text, Required
75	base_nsaids_dose<br>Show the field ONLY if:<br>[base_nsaids] = '1' or [base_nsaids] = '2'	NSAIDs total daily dose (mg)<br>*If unknown use 9999.*	text (number, Min: 0, Max: 9999), Required
76	base_paracetamol	Paracetamol	yesno, Required<br>1 Yes<br>0 No
77	base_other_tx	If any other relevant medication (i.e. Immunosuppressive therapy,) specify which<br>*Use a comma to separate the diagnoses. Example: ezetimibe, levothyroxine.*	notes
78	base_lvef	Left ventricular ejection fraction (LVEF) (%)<br>Assessed by any method.<br>*If unknow use 999.*	text (number, Min: 0, Max: 999), Required
79	base_vhd_mod_sev	Did the patient have any Moderate or Severe valvular heart disease?<br>*Example: aortic stenosis, aortic regurgitation, Mitral regurgitation, Tricuspid regurgitation, etc.*	yesno, Required<br>1 Yes<br>0 No
80	base_vhd_mod_sev_1<br>Show the field ONLY if:<br>[base_vhd_mod_sev] = '1'	Specify which type of Moderate or severe valvular heart disease	radio, Required<br>1 Aortic stenosis<br>2 Aortic regurgitation<br>3 Mitral regurgitation<br>4 Mitral Stenosis<br>5 Tricuspid regurgitation
81	base_vhd_mod_sev_2<br>Show the field ONLY if:<br>[base_vhd_mod_sev] = '1'	If the patient had a second Moderate or severe valvular heart disease, specify which type	radio<br>1 Aortic stenosis<br>2 Aortic regurgitation<br>3 Mitral regurgitation<br>4 Mitral Stenosis<br>5 Tricuspid regurgitation
82	base_valve_replacement	Did the patient have any type of previous valve repair or replacement?<br>*Surgical or transcatheter valve replacement.*	yesno, Required<br>1 Yes<br>0 No
83	base_valve_replace_type	Which type of valve repair or replacement?	radio, Required

	Show the field ONLY if: [base_valve_replacement] = '1 '		1 Transcatheter valve replacement<br>2 Surgical valve replacement<br>3 Surgical valve repair<br>4 Transcatheter and Surgical valve replacement
84	base_valve_replaced<br>Show the field ONLY if: [base_valve_replacement] = '1 '	Which cardiac valve was repair or replaced?	radio, Required<br>1 Aortic<br>2 Mitral<br>3 Tricuspid<br>4 Aortic and Mitral<br>5 Aortic, Mitral, and Tricuspid<br>6 Other
85	base_valve_replaced_other<br>Show the field ONLY if: [base_valve_replaced] = '6'	If other cardiac valve was replaced, specify which	text, Required
86	base_cci	Section Header: *BASELINE - COMORBIDITY AND FRAILTY*<br>Calculate the Charlson Comorbidity Index (CCI) and provide the numerical value (minimum 0 maximum 37)	text (number, Min: 0, Max: 50), Required
87	base_cci_weblink	Online Charlson Comorbidity index calculator (https://www.mdcalc.com/charlson-comorbidity-index-cci)	descriptive
88	base_frailty	Patient functional health status	radio, Required<br>0 Independent<br>1 Partially dependent<br>2 Totally dependent
89	baseline_complete	Section Header: *Form Status*<br>Complete?	dropdown<br>0 Incomplete<br>1 Unverified<br>2 Complete

Instrument: **COVID-19 status & Symptoms** (covid19_status_symptoms)

90	covid_swab	Section Header: *COVID-19 STATUS*<br>Coronavirus swab result	radio, Required<br>0 Negative<br>1 Positive
91	covid_swab_date	Coronavirus swab date<br>*Date in which the coronavirus swab was performed.*	text (date_dmy, Min: 2020-02-01), Required
92	covid_other_virus<br>Show the field ONLY if: [covid_swab] = 0	If swab negative for coronavirus, specify if there was any other respiratory virus.	radio, Required<br>0 No<br>1 Influenza A<br>2 Influenza B<br>3 Human respiratory syncytial virus
93	hospitalization	Section Header: *NEED OF HOSPITAL ADMISSION*<br>Did the patient require hospital admission?<br>*In any moment.*	radio, Required<br>0 No - (Ambulatory care in subjects home)<br>1 Yes - (Emergency department, hospital admission, health hotel, etc.)
94	covid_antibody_test	Section Header: *COVID-19 ANTIBODY TEST*<br>Did the patient perform a Coronavirus antibody test?<br>*Serology test, any type.*	yesno, Required<br>1 Yes<br>0 No
95	covid_antibody_date<br>Show the field ONLY if: [covid_antibody_test] = '1'	If the patient performed a Coronavirus antibody test, indicate the date.	text (date_dmy, Min: 2020-02-01), Required
96	covid_antibody_igg<br>Show the field ONLY if: [covid_antibody_test] = '1'	If the patient performed an antibody test, provide the result of IgG.	radio<br>0 Negative<br>1 Positive

97	covid_antibody_igm<br>Show the field ONLY if:<br>[covid_antibody_test] = '1'	If the patient performed an antibody test, provide the result of IgM.	radio<br>0 Negative<br>1 Positive
98	covid_antibody_iga<br>Show the field ONLY if:<br>[covid_antibody_test] = '1'	If the patient performed an antibody test, provide the result of IgA.	radio<br>0 Negative<br>1 Positive
99	covid_symptons	Section Header: *PATIENT SYMPTOMS*<br>Did the patient present any suspected viral disease symptoms?<br>*Example: fever, dyspnea, cough, diarrhea, anosmia/ageusia, sore throat, cephalea, etc. Choose NO only when the patient was completely asymptomatic.*	yesno, Required<br>1 Yes<br>0 No
100	covid_symptons_date<br>Show the field ONLY if:<br>[covid_symptons] = '1'	Beginning of symptoms	text (date_dmy, Min: 2020-01-01), Required
101	symp_fever<br>Show the field ONLY if:<br>[covid_symptons] = '1'	Fever (>37.3ºC)	yesno, Required<br>1 Yes<br>0 No
102	symp_dysnea<br>Show the field ONLY if:<br>[covid_symptons] = '1'	Dyspnea	yesno, Required<br>1 Yes<br>0 No
103	symp_cough<br>Show the field ONLY if:<br>[covid_symptons] = '1'	Cough	radio, Required<br>1 No<br>2 Yes without sputum<br>3 Yes with sputum
104	symp_diarrhea<br>Show the field ONLY if:<br>[covid_symptons] = '1'	Diarrhea	yesno, Required<br>1 Yes<br>0 No
105	symp_anosmia<br>Show the field ONLY if:<br>[covid_symptons] = '1'	Anosmia or Ageusia	yesno, Required<br>1 Yes<br>0 No
106	covid19_status_symptoms_complete	Section Header: *Form Status*<br>Complete?	dropdown<br>0 Incomplete<br>1 Unverified<br>2 Complete

Instrument: **Hospitalization** (hospitalization) Collaps

107	inhosp_adm_date<br>Show the field ONLY if:<br>[hospitalization] = '1'	Section Header: *HOSPITALIZATION (including (Emergency department, hospital admission, and health hotel).*<br>Hospital admission date	text (date_dmy, Min: 2020-02-01), Required
108	inhosp_covid_admission<br>Show the field ONLY if:<br>[hospitalization] = '1'	Was hospital admission related to COVID-19?<br>*Including emergency department, hospital admission, health hotel, etc.*	yesno, Required<br>1 Yes<br>0 No
109	inhosp_cv_disease<br>Show the field ONLY if:<br>[inhosp_covid_admission] = '0'	Was the patient admitted to the hospital because of cardiovascular disease?	yesno, Required<br>1 Yes<br>0 No
110	inhosp_cv_diag<br>Show the field ONLY if:<br>[inhosp_cv_disease] = '1'	Specify which cardiovascular disease (including pulmonary embolism)?	dropdown, Required<br>1 STEMI<br>2 NSTEMI<br>3 Chest pain/angor (negative troponin)<br>4 Heart failure<br>5 Cardiogenic Shock<br>6 Supraventricular Arrhythmia

			[illegible]<br>7 Ventricular Arrhythmia<br>8 Syncope<br>9 Cardiac arrest<br>10 Pulmonary embolism<br>11 Aortic dissection<br>12 Endocarditis<br>13 Other
111	inhosp_cv_diag_other<br>Show the field ONLY if:<br>[inhosp_cv_diag] = '13'	If other, specify which cardiovascular disease.	text, Required
112	inhosp_noncv_adm_specify<br>Show the field ONLY if:<br>[inhosp_cv_disease] = '0'	If the patient was the patient admitted with a non-cardiovascular diagnosis, specify which (DIAGNOSIS, SPECIALTY) and if any SURGERY was needed?<br>*Use a comma to separate. Example: hip fracture, trauma, hip replacement.*	notes
113	inhosp_icu_semi<br>Show the field ONLY if:<br>[hospitalization] = '1'	Did the patient need intensive or semi-intensive care unit admission?	yesno, Required<br>1 Yes<br>0 No
114	inhosp_icu_semi_admin<br>Show the field ONLY if:<br>[inhosp_icu_semi] = '1'	If intensive or semi-intensive was needed, specify where.<br>*If the patient was admitted in both, choose the Intensive care Unit.*	radio, Required<br>1 Semi-Intensive Care Units<br>2 Intensive care Unit
115	inhosp_icu_adm_date<br>Show the field ONLY if:<br>[inhosp_icu_semi] = '1'	Intensive Care Unit admission date	text (date_dmy, Min: 2020-02-01), Required
116	inhosp_icu_disch_date<br>Show the field ONLY if:<br>[inhosp_icu_semi] = '1'	Intensive care Unit discharge date	text (date_dmy, Min: 2020-02-01), Required
117	inhosp_imv<br>Show the field ONLY if:<br>[inhosp_icu_semi] = '1'	Invasive mechanical ventilation	yesno, Required<br>1 Yes<br>0 No
118	inhosp_ecmo<br>Show the field ONLY if:<br>[inhosp_icu_semi] = '1'	Extracorporeal membrane oxygenation (ECMO)	radio, Required<br>0 No<br>1 ECMO V-V<br>2 ECMO V-A
119	inhosp_rrt<br>Show the field ONLY if:<br>[hospitalization] = '1'	Renal replacement therapy	yesno, Required<br>1 Yes<br>0 No
120	inhosp_vasodrug<br>Show the field ONLY if:<br>[hospitalization] = '1'	Need of vasoactive agents<br>*Example: dopamine, dobutamine, noradrenaline*	yesno, Required<br>1 Yes<br>0 No
121	inhosp_lvef<br>Show the field ONLY if:<br>[hospitalization] = '1'	Left-ventricular ejection fraction (%)<br>*Assessed by any method. If unknown use 999.*	text (number, Min: 0, Max: 999), Required
122	inhosp_cath_available<br>Show the field ONLY if:<br>[hospitalization] = '1'	Section Header: *CARDIAC CATHETERIZATION FINDINGS*<br>Was a cardiac catheterization performed?<br>*PCI: percutaneous cardiac intervention*	yesno, Required<br>1 Yes<br>0 No
123	inhosp_cath_pain_date<br>Show the field ONLY if:<br>[inhosp_cath_available] = '1'	Date of the cardiac catheterization	text (date_dmy, Min: 2020-02-01), Required
124	inhosp_cath_indication<br>Show the field ONLY if:<br>[inhosp_cath_available] = '1'	Cardiac catheterization clinical indication	radio, Required<br>1 Angina pectoris (troponin negative)<br>2 Unstable angina (troponin negative)

			3 NSTEMI<br>4 STEMI
125	inhosp_cath_culprit<br><br>Show the field ONLY if:<br>[inhosp_cath_available] = '1'	Was there a culprit lesion?	radio, Required<br>0 No<br>1 Yes - No thrombotic<br>2 Yes - Thrombotic
126	inhosp_cath_coro_seg<br><br>Show the field ONLY if:<br>[inhosp_cath_available] = '1'	Choose the culprit lesion coronary segment	radio<br>1 Left main coronary artery<br>2 Left anterior descending artery<br>3 Left circumflex artery<br>4 Right coronary artery
127	inhosp_cath_mv<br><br>Show the field ONLY if:<br>[inhosp_cath_available] = '1'	Multi-vessel disease	yesno, Required<br>1 Yes<br>0 No
128	inhosp_cath_coro_seg_non1<br><br>Show the field ONLY if:<br>[inhosp_cath_mv] = '1'	If multi-vessel disease, choose the non-culprit lesion #1 coronary segment	radio<br>1 Left main coronary artery<br>2 Left anterior descending artery<br>3 Left circumflex artery<br>4 Right coronary artery
129	inhosp_cath_coro_seg_non2<br><br>Show the field ONLY if:<br>[inhosp_cath_mv] = '1'	If multi-vessel disease, choose the non-culprit lesion #2 coronary segment	radio<br>1 Left main coronary artery<br>2 Left anterior descending artery<br>3 Left circumflex artery<br>4 Right coronary artery
130	inhosp_pci<br><br>Show the field ONLY if:<br>[hospitalization] = '1'	Was Percutaneous coronary intervention (PCI) performed?<br>*Example: thrombus aspiration, stent implantation, drug-eluting balloon.*	yesno, Required<br>1 Yes<br>0 No
131	inhosp_cath_asa_ld<br><br>Show the field ONLY if:<br>[inhosp_pci] = '1'	Aspirin loading dose?	yesno, Required<br>1 Yes<br>0 No
132	inhosp_cath_p2y12_ld<br><br>Show the field ONLY if:<br>[inhosp_pci] = '1'	P2Y12 inhibitor loading dose?<br>*If the patient was loaded with clopidogrel and thereafter was switch to Prasugrel or Ticagrelor, select the latter.*	radio, Required<br>0 No<br>1 Clopidogrel<br>2 Prasugrel<br>3 Ticagrelor
133	inhosp_cath_anticoag<br><br>Show the field ONLY if:<br>[inhosp_pci] = '1'	Anticoagulation treatment during the procedure?	radio, Required<br>0 No<br>1 Unfractioned heparin<br>2 Bivalirudin
134	inhosp_pci_gpi<br><br>Show the field ONLY if:<br>[inhosp_pci] = '1'	Was GPI used (IIB/IIIA inhibitors)?	yesno, Required<br>1 Yes<br>0 No
135	inhosp_cath_device<br><br>Show the field ONLY if:<br>[inhosp_pci] = '1'	Device type	radio, Required<br>0 Trombus aspiration only<br>1 Bare-metal stent<br>2 Drug-eluting stent<br>3 Drug-eluting balloon
136	inhosp_cath_device_brand	Please provide the device commercial name<br>*Example: xience, onyx, orsiro, etc*	text, Required

	Show the field ONLY if:<br>[inhosp_cath_device] = '1' or [inhosp_cath_device] = '2' or [inhosp_cath_device] = '3'	[illegible]	
137	inhosp_pci_compli<br>Show the field ONLY if:<br>[inhosp_cath_available] = '1'	Describe any complication during the procedure:<br>1.- Site access hematoma/bleeding<br>2.- Coronary dissection/perforation<br>3.- Acute Stent thrombosis<br>*Use a comma to separate the complications.*	notes
138	inhosp_cath_ventri<br>Show the field ONLY if:<br>[inhosp_cath_available] = '1'	Was left ventriculography performed?	yesno, Required<br>1 Yes<br>0 No
139	inhosp_cath_ventri_tako<br>Show the field ONLY if:<br>[inhosp_cath_ventri] = '1'	If ventriculography performed, was there any Takotsubo pattern?<br>*Any pattern (Apical, mid ventricular, basal or focal)*<br>*https://takotsubo.net/information/about-takotsubo-syndrome/*	yesno, Required<br>1 Yes<br>0 No
140	inhosp_cath_second<br>Show the field ONLY if:<br>[inhosp_cath_available] = '1'	If the patient had a Second cardiac catheterization (for example, stent thrombosis or revascularization), specify the following:<br>Date, diagnosis, procedure, and outcome:<br>*Use a comma to separate the date, diagnosis, procedure, and outcome.*	notes
141	inhosp_thrombo<br>Show the field ONLY if:<br>[hospitalization] = '1'	Section Header: *IN-HOSPITAL THROMBOLYSIS THERAPY*<br>Was thrombolysis therapy performed?	yesno, Required<br>1 Yes<br>0 No
142	inhosp_thrombo_why<br>Show the field ONLY if:<br>[inhosp_thrombo] = '1'	If thrombolysis was performed, which was the clinical indication?	radio, Required<br>1 ST-segment elevation Myocardial infarction<br>2 Ischemic stroke<br>3 Pulmonary embolism
143	inhosp_anticoag<br>Show the field ONLY if:<br>[hospitalization] = '1'	Section Header: *IN-HOSPITAL ANTICOAGULANT TREATMENT*<br>Was the patient treated with any anticoagulant therapy?<br>*Example: acenocoumarol, enoxaparin, rivaroxaban, unfractionated heparin, etc.*	yesno, Required<br>1 Yes<br>0 No
144	inhosp_anticoag_type<br>Show the field ONLY if:<br>[inhosp_anticoag] = '1'	Which type of Anticoagulant treatment?<br>**Example: Rivaroxaban, dabigatran, apixaban, or edoxaban.*	radio, Required<br>1 Vitamin K antagonists (acenocoumarol)<br>2 Directly acting oral anticoagulants*<br>3 Low-molecular-weight heparin (Enoxaparin, et[illegible]<br>4 Unfractionated heparin (UFH) (Sodic)
145	inhosp_anticoag_type_2nd<br>Show the field ONLY if:<br>[inhosp_anticoag] = '1'	If the patient was treated with a second anticoagulant treatment, specify which<br>**Example: Rivaroxaban, dabigatran, apixaban, or edoxaban.*	radio<br>1 Vitamin K antagonists (acenocoumarol)<br>2 Directly acting oral anticoagulants*<br>3 Low-molecular-weight heparin (Enoxaparin, et[illegible]<br>4 Unfractionated heparin (UFH) (Sodic)
146	inhosp_anticoagulant_why<br>Show the field ONLY if:<br>[inhosp_anticoag] = '1'	Clinical indication for anticoagulant treatment	radio, Required<br>1 Prophylaxis<br>2 Atrial fibrillation<br>3 Acute coronary syndrome<br>4 Venous thromboembolism<br>5 Pulmonary embolism<br>6 Arterial embolism<br>7 Stroke<br>8 Cardiac intraventricular thrombus<br>9 Other
147	inhosp_anticoag_other<br>Show the field ONLY if:<br>[inhosp_anticoagulant_why] = '9'	If other indication for anticoagulant treatment, specify which	text, Required

148	inhosp_anticoagulant_why_2<br>Show the field ONLY if:<br>[inhosp_anticoag] = '1'	If there was a second clinical indication for anticoagulant treatment, specify which	radio<br>1 Prophylaxis<br>2 Atrial fibrillation<br>3 Acute coronary syndrome<br>4 Venous thromboembolism<br>5 Pulmonary embolism<br>6 Arterial embolism<br>7 Stroke<br>8 Cardiac intraventricular thrombus
149	inhosp_lwmh_tx_type<br>Show the field ONLY if:<br>[inhosp_anticoag_type] = 3 or [inhosp_anticoag_type_2nd] = 3	Which Low-molecular-weight heparin was used?	radio, Required<br>1 Enoxaparin<br>2 Dalteparin<br>3 Bemiparin<br>4 Tinzaparin<br>5 Fondaparinux<br>6 Nadroparin<br>7 Certoparin<br>8 Other
150	inhosp_lmwh_other<br>Show the field ONLY if:<br>[inhosp_lwmh_tx_type] = '8'	If other, please specify the type of Low-molecular-weight heparin:	text, Required
151	inhosp_lmwh_dose<br>Show the field ONLY if:<br>[inhosp_anticoag_type] = 3 or [inhosp_anticoag_type_2nd] = 3	Specify the highest dose of Low-molecular-weight heparin (only numbers) that was used.<br>*Example: 40 mg or 4000 UI (only numbers).*	text (number, Min: 0, Max: 10000000), Required
152	inhosp_lmwh_unit<br>Show the field ONLY if:<br>[inhosp_anticoag_type] = 3 or [inhosp_anticoag_type_2nd] = 3	Which was the Low-molecular-weight heparin UNIT? (mg or UI)<br>*mg or UI*	radio, Required<br>1 mg<br>2 UI
153	inhosp_lmwh_frequency<br>Show the field ONLY if:<br>[inhosp_anticoag_type] = 3 or [inhosp_anticoag_type_2nd] = 3	Which was the frequency of administration of the Low-molecular-weight heparin?	radio, Required<br>1 Each 24 hours<br>2 Each 12 hours
154	inhosp_high_dose_date<br>Show the field ONLY if:<br>[coordinating_center] = '1' and [inhosp_anticoag_type] = 3 or [inhosp_anticoag_type_2nd] = 3	What day was the highest dose of low-weight molecular heparin started?	text (date_dmy, Min: 2020-02-01), Required
155	inhosp_lmwh_days<br>Show the field ONLY if:<br>[coordinating_center] = '1' and [inhosp_anticoag_type] = 3 or [inhosp_anticoag_type_2nd] = 3	TOTAL days of low-weight molecular heparin treatment	text (number, Min: 0, Max: 100), Required
156	inhosp_noac_type<br>Show the field ONLY if:<br>[inhosp_anticoag_type] = 2 or [inhosp_anticoag_type_2nd] = 2	If Directly acting oral anticoagulants were used, specify which	radio, Required<br>1 Rivaroxaban (Xarelto)<br>2 Dabigatran (Pradaxa)<br>3 Apixaban (Eliquis)<br>4 Edoxaban (Lixiana)
157	inhosp_noac_dose	Specify the Directly acting oral anticoagulants TOTAL Daily dose (mg)	text (number, Min: 0, Max: 999), Required

	Show the field ONLY if: [inhosp_anticoag_type] = 2 or [inhosp_anticoag_type_2nd] = 2	*If unknown use 999.*	
158	inhosp_aspirin<br>Show the field ONLY if: [hospitalization] = '1'	Section Header: *IN-HOSPITAL TREATMENT (If data is not available, select not available option)*<br>Aspirin	radio, Required<br>0 No<br>1 Yes<br>2 Not available
159	inhosp_p2y12<br>Show the field ONLY if: [hospitalization] = '1'	P2Y12 inhibitor<br>*Example: clopidogrel, prasugrel, Ticagrelor*	radio, Required<br>0 No<br>1 Yes<br>2 Not available
160	inhosp_p2y12_type<br>Show the field ONLY if: [inhosp_p2y12] = '1'	Which P2Y12 inhibitor	radio, Required<br>1 Clopidogrel<br>2 Prasugrel<br>3 Ticagrelor<br>4 Not available
161	inhosp_ace_inh<br>Show the field ONLY if: [hospitalization] = '1'	ACE inhibitor<br>*Angiotensin-converting-enzyme inhibitors (ACE inhibitors)*	radio, Required<br>0 No<br>1 Yes<br>2 Not available
162	inhosp_arbs<br>Show the field ONLY if: [hospitalization] = '1'	ARBs<br>*Angiotensin II receptor blockers*	radio, Required<br>0 No<br>1 Yes<br>2 Not available
163	inhosp_statins<br>Show the field ONLY if: [hospitalization] = '1'	Statins	radio, Required<br>0 No<br>1 Yes<br>2 Not available
164	inhosp_beta_block<br>Show the field ONLY if: [hospitalization] = '1'	Beta Blocker	radio, Required<br>0 No<br>1 Yes<br>2 Not available
165	inhosp_ccb<br>Show the field ONLY if: [hospitalization] = '1'	Calcium channel blocker	radio, Required<br>0 No<br>1 Yes<br>2 Not available
166	inhosp_loop_diuretics<br>Show the field ONLY if: [hospitalization] = '1'	Loop diuretic<br>*Example: Furosemide, Torsemide, etc.*	radio, Required<br>0 No<br>1 Yes<br>2 Not available
167	inhosp_mras<br>Show the field ONLY if: [hospitalization] = '1'	Mineralocorticoid receptor antagonists (MRAs)<br>*Spironolactone or eplerenone*	radio, Required<br>0 No<br>1 Yes<br>2 Not available
168	inhosp_thrombofilia_study<br>Show the field ONLY if: [coordinating_center] = '1' and [hospitalization] = '1'	Section Header: *IN-HOSPITAL - THROMBOPHILIA AND/OR AUTOINMMUNITY STUDY*<br>Was thrombophilia study performed to the patient?	yesno, Required<br>1 Yes<br>0 No
169	inhosp_thrombofilia_lab	If a thrombophilia study was done, indicate the date of the	text (date_dmy, Min: 2020-02-01), Required

	Show the field ONLY if: [inhosp_thrombofilia_study] = '1'	laboratory.	
170	inhosp_autoimmunity_study<br>Show the field ONLY if: [coordinating_center] = '1' an d [hospitalization] = '1'	Was autoimmunity study performed to the patient?	yesno, Required<br>1 Yes<br>0 No
171	inhosp_autoinmune_lab<br>Show the field ONLY if: [inhosp_autoimmunity_study] = '1'	If a autoinmmunity study was done, indicate the date of the laboratory.<br>*Please anonymize the report and upload it*	text (date_dmy, Min: 2020-02-01), Required
172	hospitalization_complete	Section Header: *Form Status*<br>Complete?	dropdown<br>0 Incomplete<br>1 Unverified<br>2 Complete
Instrument: **COVID-19 treatment** (covid19_treatment)			Collaps
173	covid_tx_kaletra	Section Header: *COVID-19 SPECIFIC TREATMENT (at any time or setting [ambulatory or hosptial])*<br>Kaletra (Lopinavir/Ritonavir)?	yesno, Required<br>1 Yes<br>0 No
174	covid_tx_hydroxyclor	Hydroxychloroquine?	yesno, Required<br>1 Yes<br>0 No
175	covid_tx_azithro	Azithromycin?	yesno, Required<br>1 Yes<br>0 No
176	covid_tx_toci	Tocilizumab (Actemra)?	yesno, Required<br>1 Yes<br>0 No
177	covid_tx_antibody	Did the patient was treated with a second monoclonal antibody? (Example: Canakinumab, etc.)<br>*Example: Canakinumab, Sarilumab, or Siltuximab.*	yesno, Required<br>1 Yes<br>0 No
178	covid_tx_antibody_type<br>Show the field ONLY if: [covid_tx_antibody] = '1'	Which monoclonal antibody?	radio, Required<br>1 Canakinumab (Anakinra)<br>2 Sarilumab (Kevzara)<br>3 Siltuximab (Sylvant)<br>4 Other
179	covid_tx_antibody_other<br>Show the field ONLY if: [covid_tx_antibody_type] = '4'	If other, specify which monoclonal antibody	text, Required
180	covid_tx_cortis	Corticosteroids<br>*Example: Methylprednisolone, prednisone, dexamethasone, etc.*	yesno, Required<br>1 Yes<br>0 No
181	covid_tx_cortis_type<br>Show the field ONLY if: [covid_tx_cortis] = '1'	Specify which type of Corticosteroids	radio, Required<br>1 Methylprednisolone<br>2 Dexamethasone<br>3 Prednisone<br>4 Prednisolone<br>5 Hydrocortisone<br>6 Other
182	covid_tx_cortis_other	If other corticosteroids, specify which:	text, Required

	Show the field ONLY if:<br>[covid_tx_cortis_type] = '6'		
183	covid_tx_cortis_dose<br><br>Show the field ONLY if:<br>[covid_tx_cortis] = '1'	Which was the maximum used dose of corticosteroids (mg)?<br>*Example: 250 mg (only numbers).*	text, Required
184	covid_tx_remde	Remdesivir?	yesno, Required<br>1 Yes<br>0 No
185	inhosp_nsaids	Nonsteroidal anti-inflammatory drugs (NSAIDs)	yesno, Required<br>1 Yes<br>0 No
186	covid_nsaid_type<br><br>Show the field ONLY if:<br>[inhosp_nsaids] = '1'	If non steroidal anti inflammatory (NSAIDs) were use, specify which type	radio, Required<br>1 Ibuprofen<br>2 Diclofenac<br>3 Naproxen<br>4 Dexketoprofen or ketoprofen<br>5 Celecoxib<br>6 Other
187	inhosp_nsaids_other<br><br>Show the field ONLY if:<br>[covid_nsaid_type] = '6'	If other non steroidal anti inflammatory (NSAIDs), specify which	text, Required
188	covid_paracetamol	Paracetamol	yesno, Required<br>1 Yes<br>0 No
189	covid_tx_other	If there was any other COVID-19 specify treatment, specify:<br>*Use a comma to separate.*	notes
190	covid19_treatment_complete	Section Header: *Form Status*<br><br>Complete?	dropdown<br>0 Incomplete<br>1 Unverified<br>2 Complete

Instrument: **Biomarkers** (biomarkers) Collaps

191	inhosp_ctni	Section Header: *INDEX EVALUATION - CARDIAC BIOMARKERS (In-hospital, emergency department, or ambulatory)*<br><br>Was the patient tested for Troponin I?	yesno, Required<br>1 Yes<br>0 No
192	inhosp_cnti_positive_adm<br><br>Show the field ONLY if:<br>[inhosp_ctni] = '1'	Was Troponin I already elevated in the first test?	yesno, Required<br>1 Yes<br>0 No
193	inhosp_ctni_peak<br><br>Show the field ONLY if:<br>[inhosp_ctni] = '1'	Hs–cardiac Troponin I peak<br>*Highest value (numerical) during the index hospitalization or ambulatory.*	text (number, Min: 0, Max: 10000000), Required
194	inhosp_ctni_unit<br><br>Show the field ONLY if:<br>[inhosp_ctni] = '1'	Hs–cTn–I - units	dropdown, Required<br>1 ng/L<br>2 ng/mL<br>3 µg/L<br>4 pg/mL<br>5 ng/dL
195	inhosp_ctni_peak_date<br><br>Show the field ONLY if:<br>[inhosp_ctni] = '1'	Hs–cTn–I peak date	text (date_dmy, Min: 2020-02-24), Required
196	inhosp_ctnt	Was the patient tested for Troponin T?	yesno, Required<br>1 Yes

			0 No
197	inhosp_cntt_positive_adm<br>Show the field ONLY if:<br>[inhosp_ctnt] = '1'	Was Troponin T already elevated in the first test?	yesno, Required<br>1 Yes<br>0 No
198	inhosp_ctnt_peak<br>Show the field ONLY if:<br>[inhosp_ctnt] = '1'	Hs–cTn–T peak<br>*Highest value (numerical) during the index hospitalization or ambulatory.*	text (number, Min: 0, Max: 10000000), Required
199	inhosp_ctnt_unit<br>Show the field ONLY if:<br>[inhosp_ctnt] = '1'	Hs–cTn–T - units	dropdown, Required<br>1 ng/L<br>2 ng/mL<br>3 μg/L<br>4 pg/mL<br>5 ng/dL
200	inhosp_ctnt_peak_date<br>Show the field ONLY if:<br>[inhosp_ctnt] = '1'	Hs–cTn–T peak date	text (date_dmy, Min: 2020-02-24), Required
201	carbio_bnp	Was the patient tested for B-type natriuretic peptide (BNP) or NT-BNP?	yesno, Required<br>1 Yes<br>0 No
202	inhosp_pronbp_peak<br>Show the field ONLY if:<br>[carbio_bnp] = 1	NT-proBNP peak (pg/mL)<br>*Highest value (numerical) during the index hospitalization or ambulatory.*	text (number, Min: 0, Max: 10000000), Required
203	inhosp_bnp_peak<br>Show the field ONLY if:<br>[carbio_bnp] = 1	B-type natriuretic peptide peak (pg/mL)<br>*Highest value (numerical) during the index hospitalization or ambulatory.*	text (number, Min: 0, Max: 10000000), Required
204	inhosp_ddimer	Was the patient tested for D dimer?	yesno, Required<br>1 Yes<br>0 No
205	inhosp_ddimer_peak<br>Show the field ONLY if:<br>[inhosp_ddimer] = '1'	D-dimer (ng/mL) peak<br>*Highest value (numerical) during the index hospitalization or ambulatory.*	text (number, Min: 0, Max: 10000000), Required
206	inhosp_ddimer_peak_date<br>Show the field ONLY if:<br>[inhosp_ddimer] = '1'	D-dimer peak date	text (date_dmy, Min: 2020-02-24), Required
207	inhosp_pt<br>Show the field ONLY if:<br>[coordinating_center] = '1'	Was the patient tested for prothrombin time?	yesno, Required<br>1 Yes<br>0 No
208	inhosp_pt_peak<br>Show the field ONLY if:<br>[inhosp_pt] = '1'	Prothrombin time (seconds) peak:<br>*Highest value (numerical) during the index hospitalization or ambulatory.*	text (number, Min: 0, Max: 300), Required
209	inhosp_pt_peak_date_2<br>Show the field ONLY if:<br>[inhosp_pt] = '1'	Prothrombin time (seconds) peak date:	text (date_dmy, Min: 2020-02-24), Required
210	inhosp_fibri<br>Show the field ONLY if:<br>[coordinating_center] = '1'	Was the patient tested for fibrinogen?	yesno, Required<br>1 Yes<br>0 No
211	inhosp_fibri_lowest<br>Show the field ONLY if:<br>[inhosp_fibri] = '1'	Fibrinogen lowest value (g/L):<br>*Lowest value (numerical) during the index hospitalization or ambulatory.*	text (number, Min: 0, Max: 10), Required
212	inhosp_fibri_lowest_date<br>Show the field ONLY if:	Fibrinogen lowest value (g/L) date:	text (date_dmy, Min: 2020-02-24), Required

	[inhosp_fibri] = '1'		
213	inhosp_hb_low	Section Header: *IN-HOSPITAL - BLOOD COUNT (If Hemoglobin or platelet values are unknown use 999).*<br>Hemoglobin (g/L) lowest value<br>*Lowest value (numerical) during the index hospitalization or ambulatory. If unknown use 999.*	text (number, Min: 0, Max: 999), Required
214	inhosp_hb_lowest_date<br>Show the field ONLY if:<br>[coordinating_center] = '1' and [inhosp_hb_low] < '999'	Hemoglobin (g/L) lowest date	text (date_dmy, Min: 2020-02-24), Required
215	inhosp_plt_low	Platelet (10x9/L) lowest value<br>*Lowest value (numerical) during the index hospitalization or ambulatory. If unknown use 999.*	text (number, Min: 0, Max: 999), Required
216	inhosp_plt_lowest_date<br>Show the field ONLY if:<br>[coordinating_center] = '1' and [inhosp_plt_low] < '999'	Platelet (10x9/L) lowest date	text (date_dmy, Min: 2020-02-24), Required
217	inhosp_lymph	Lymphocyte count (10x9/L) lowest value<br>*Lowest value (numerical) during the index hospitalization or ambulatory. If unknown use 999.*	text (number, Min: 0, Max: 999), Required
218	inhosp_plt_lowest_date_2<br>Show the field ONLY if:<br>[coordinating_center] = '1' and [inhosp_lymph] < '999'	Lymphocyte count (10x9/L) lowest value date	text (date_dmy, Min: 2020-02-24), Required
219	inhosp_cr_peak	Section Header: *IN-HOSPITAL - SERUM BIOCHEMISTRY (If Creatinine or C-reactive protein values are unknown use 999).*<br>Creatinine (mg/dL) highest peak<br>*Highest value (numerical) during the index hospitalization or ambulatory. If unknown use 999.*	text (number, Min: 0, Max: 999), Required
220	inhosp_cr_peak_date<br>Show the field ONLY if:<br>[coordinating_center] = '1' and [inhosp_cr_peak] < '999'	Creatinine (mg/dL) highest peak date	text (date_dmy, Min: 2020-02-24), Required
221	inhosp_crp_peak	C-reactive protein (CRP) (mg/dL) peak value<br>*Highest value (numerical) during the index hospitalization or ambulatory. If unknown use 999.*	text (number, Min: 0, Max: 999), Required
222	inhosp_crp_peak_date<br>Show the field ONLY if:<br>[inhosp_crp_peak] < '999'	C-reactive protein (CRP) (mg/dL) peak date	text (date_dmy, Min: 2020-02-24), Required
223	biomarkers_complete	Section Header: *Form Status*<br>Complete?	dropdown<br>0 Incomplete<br>1 Unverified<br>2 Complete

Instrument: **Discharge, Medications & Outcomes** (discharge_medications_outcomes) Collaps

224	dis_discharge_date<br>Show the field ONLY if:<br>[hospitalization] = '1'	Section Header: *IN-HOSPITAL OUTCOMES*<br>Hospital discharge date<br>*If the patient dies during hospitalization, use the date of death.*	text (date_dmy, Min: 2020-02-01), Required
225	inhosp_out_mortality<br>Show the field ONLY if:<br>[hospitalization] = '1'	-- IN-HOSPITAL PATIENT MORTALITY --<br>(PRIMARY ENDPOINT)	yesno, Required<br>1 Yes<br>0 No
226	inhosp_out_mortality_date<br>Show the field ONLY if:<br>[inhosp_out_mortality] = '1'	Date of death	text (date_dmy, Min: 2020-02-01), Required
227	inhosp_mort_report<br>Show the field ONLY if:<br>[inhosp_out_mortality] = '1'	Anonymize and upload the discharge (mortality) report. Document needed for death adjudication.	file
228	inhosp_out_cv_death<br>Show the field ONLY if:<br>[inhosp_out_mortality] = '1'	Suspected cardiovascular death	radio<br>1 Death caused by acute MI<br>2 Death caused by sudden cardiac, including

	[illegible]		2 Death caused by sudden cardiac, including unwitnessed<br>3 Death resulting from heart failure<br>4 Death caused by stroke<br>5 Death caused by cardiovascular procedures<br>6 Death resulting from cardiovascular hemorrha<br>7 Death resulting from other cardiovascular caus
229	inhosp_out_noncv_death<br>Show the field ONLY if:<br>[inhosp_out_mortality] = '1'	Suspected Non-cardiovascular death	radio<br>1 Death resulting from malignancy<br>2 Death resulting from pulmonary causes<br>3 Death caused by infection (includes sepsis)<br>4 Death resulting from gastrointestinal causes<br>5 Death resulting from accident/trauma<br>6 Death caused by other noncardiovascular orga failure<br>7 Death resulting from other noncardiovascular cause<br>8 Undetermined cause of death
230	inhosp_out_acs<br>Show the field ONLY if:<br>[hospitalization] = '1'	IN-HOSPITAL ACS?<br>(Acute Coronary Syndrome)	yesno, Required<br>1 Yes<br>0 No
231	inhosp_out_acs_type<br>Show the field ONLY if:<br>[inhosp_out_acs] = '1'	Which type of Acute Coronary Syndrome?	radio, Required<br>1 Unstable angina<br>2 NSTEMI<br>3 STEMI
232	inhosp_out_acs_date<br>Show the field ONLY if:<br>[inhosp_out_acs] = '1'	Date of the acute coronary syndrome	text (date_dmy, Min: 2020-02-01), Required
233	inhosp_out_stroke<br>Show the field ONLY if:<br>[hospitalization] = '1'	IN-HOSPITAL Stroke/transient ischemic attack?	yesno, Required<br>1 Yes<br>0 No
234	inhosp_out_stroke_type<br>Show the field ONLY if:<br>[inhosp_out_stroke] = '1'	Which type of Stroke/transient ischemic attack?	radio, Required<br>1 Transient ischemic attack<br>2 Ischemic stroke<br>3 Hemorrhagic stroke
235	inhosp_stroke_laterality<br>Show the field ONLY if:<br>[inhosp_out_stroke_type] = '2' or [inhosp_out_stroke_type] = '3'	If the patient presented a stroke, specify the laterality	radio, Required<br>1 Right<br>2 left<br>3 Both
236	inhosp_out_stroke_vessel<br>Show the field ONLY if:<br>[inhosp_out_stroke_type] = '2'	If it was an ischemic stroke, specify the type of stroke	radio, Required<br>1 Large vessel stroke (anterior or posterior cerebral circulation, basilar artery, or vertebral artery)<br>2 Small vessel stroke (lacunar)
237	inhosp_out_stroke_bleed<br>Show the field ONLY if:<br>[inhosp_out_stroke_type] = '3'	If it was a hemorrhagic (intracranial bleeding), specify the type	radio<br>1 Epidural<br>2 Subdural<br>3 Subarachnoid<br>4 Intracerebral (Intraparenchymal)<br>5 Intraventricular
238	inhosp_out_stroke_date	Date of the stroke/transient ischemic attack	text (date_dmy, Min: 2020-02-01), Required

	Show the field ONLY if: [inhosp_out_stroke] = '1'		
239	inhosp_out_vte<br>Show the field ONLY if: [hospitalization] = '1'	IN-HOSPITAL Venous/arterial thromboembolism?	yesno, Required<br>1 Yes<br>0 No
240	inhosp_out_vte_type<br>Show the field ONLY if: [inhosp_out_vte] = '1'	If the patient presented venous/arterial thromboembolism, specify which type	radio, Required<br>1 Deep vein thrombosis<br>2 Arterial embolism
241	inhosp_out_pe_vte_date<br>Show the field ONLY if: [inhosp_out_vte] = '1'	Date of venous/arterial thromboembolism	text (date_dmy, Min: 2020-02-01), Required
242	inhosp_out_vte_describe<br>Show the field ONLY if: [inhosp_out_vte] = '1'	If the patient presented a venous/arterial thromboembolism, describe the location and if invasive treatment was needed.	notes
243	inhosp_out_pe<br>Show the field ONLY if: [hospitalization] = '1'	IN-HOSPITAL Pulmonary embolism<br>*Definition requires confirmation by Thorax CT scan.*	yesno, Required<br>1 Yes<br>0 No
244	inhosp_out_pe_laterality<br>Show the field ONLY if: [inhosp_out_pe] = '1'	If the patient had a Pulmonary embolism, specify the laterality	radio, Required<br>1 Right<br>2 left<br>3 Both
245	inhosp_out_pe_vessel<br>Show the field ONLY if: [inhosp_out_pe] = '1'	If the patient had a Pulmonary embolism, specify which type	radio, Required<br>1 Major vessel (Main artery, Lobar artery, Segmental)<br>2 Minor vessel (Sub-segmental)
246	inhosp_out_pe_date<br>Show the field ONLY if: [inhosp_out_pe] = '1'	Date of Pulmonary embolism	text (date_dmy, Min: 2020-02-01), Required
247	inhosp_out_bleeding<br>Show the field ONLY if: [hospitalization] = '1'	IN-HOSPITAL major bleeding?	yesno, Required<br>1 Yes<br>0 No
248	inhosp_out_bleeding_type<br>Show the field ONLY if: [inhosp_out_bleeding] = '1'	If the patient presented a major bleeding, specify which type?	radio, Required<br>1 Bleeding with an Hb drop between 3-5 g/dL.<br>2 Bleeding requiring transfusion<br>3 Bleeding requiring surgical intervention<br>4 Non-fatal intracranial hemorrhage<br>5 Fatal bleeding without autopsy/imaging confirmation<br>6 Fatal bleeding with autopsy/imaging confirmation
249	inhosp_out_bleeding_date<br>Show the field ONLY if: [inhosp_out_bleeding] = '1'	Date of major bleeding	text (date_dmy, Min: 2020-02-01), Required
250	inhosp_out_bleeding_site<br>Show the field ONLY if: [inhosp_out_bleeding] = '1'	Specify the site of origin if the major bleeding:	radio, Required<br>1 Intracraneal or intraspinal<br>2 Pulmonary (hemoptysis, endotracheal tube, bronchoscopy)<br>3 Gastrointestinal (hematemesis, melena, uppe endoscopy or colonoscopy)<br>4 Genito-urinary (urine, urinary catheter, cystoscopy)<br>5 Nose (Epistaxis)<br>6 Soft tissue hematoma

			7 Solid organ hematoma<br>8 Ocular<br>9 Intra-abdominal bleeding<br>10 Retroperitoneal bleeding<br>11 Other
251	inhos_out_bleeding_other<br>Show the field ONLY if:<br>[inhosp_out_bleeding_site]=11	If other, describe the site of origin:	text, Required
252	inhosp_bleeding_descrip<br>Show the field ONLY if:<br>[inhosp_out_bleeding] = '1'	Provide a very small description of the bleeding event	notes, Required
253	inhosp_out_transfusion<br>Show the field ONLY if:<br>[hospitalization] = '1'	IN-HOSPITAL Red blood cell transfusion<br>*At least one red blood cell transfusion*	yesno, Required<br>1 Yes<br>0 No
254	inhosp_out_arrhyth<br>Show the field ONLY if:<br>[hospitalization] = '1'	IN-HOSPITAL Serious arrhythmias events	yesno, Required<br>1 Yes<br>0 No
255	inhosp_out_arrhyth_type<br>Show the field ONLY if:<br>[inhosp_out_arrhyth] = '1'	If the patient presented serious arrhythmias events, specify which	radio, Required<br>1 Bradicardya requiring iv Medication<br>2 Bradicardya requiring pacemaker<br>3 Supraventricular tachycardia requiring iv Medication<br>4 Supraventricular tachycardia requiring Cardioversion<br>5 Ventricular tachycardia requiring iv Medication<br>6 Ventricular tachycardia requiring Cardioversion
256	inhosp_out_arrhyth_date<br>Show the field ONLY if:<br>[inhosp_out_arrhyth] = '1'	Date of presentation of cardiac arrhythmia	text (date_dmy, Min: 2020-02-01), Required
257	inhosp_other_events<br>Show the field ONLY if:<br>[hospitalization] = '1'	If there was another relevant event, specify which.<br>*Use a comma to separate the complications. Example: urgent surgery, etc.*	notes
258	dis_aspirin<br>Show the field ONLY if:<br>[hospitalization] = '1' and [inhosp_out_mortality] = '0'	Section Header: *DISCHARGE - TREATMENT*<br>Aspirin	yesno, Required<br>1 Yes<br>0 No
259	dis_p2y12<br>Show the field ONLY if:<br>[hospitalization] = '1' and [inhosp_out_mortality] = '0'	P2Y12 inhibitor<br>*Example: clopidogrel, prasugrel, Ticagrelor*	yesno, Required<br>1 Yes<br>0 No
260	dis_p2y12_type<br>Show the field ONLY if:<br>[dis_p2y12] = '1'	Which P2Y12 inhibitor	radio, Required<br>1 Clopidogrel<br>2 Prasugrel<br>3 Ticagrelor
261	dis_ace_inh<br>Show the field ONLY if:<br>[hospitalization] = '1' and [inhosp_out_mortality] = '0'	ACE inhibitor<br>*Angiotensin-converting-enzyme inhibitors (ACE inhibitors)*	yesno, Required<br>1 Yes<br>0 No
262	dis_ace_type<br>Show the field ONLY if:<br>[dis_ace_inh] = '1'	ACE inhibitor type	radio, Required<br>1 Lisinopril<br>2 Enalapril<br>3 Perindopril

			3 Perindopril<br>4 Other
263	dis_ace_other<br>Show the field ONLY if:<br>[dis_ace_type] = '4'	If other ACE inhibitor, specify which	text, Required
264	dis_ace_dose<br>Show the field ONLY if:<br>[dis_ace_inh] = '1'	ACE inhibitor TOTAL daily dose (mg)<br>*If unknown use 999.*	text (number, Min: 1, Max: 999), Required
265	dis_arbs<br>Show the field ONLY if:<br>[hospitalization] = '1' and [inhosp_out_mortality] = '0'	ARBs<br>*Angiotensin II receptor blockers*	yesno, Required<br>1 Yes<br>0 No
266	dis_arbs_type<br>Show the field ONLY if:<br>[dis_arbs] = '1'	ARBs type	radio, Required<br>1 Losartan<br>2 Candesartan<br>3 Irbesartan<br>4 Telmisartan<br>5 Valsartan<br>6 Olmesartan<br>7 Other
267	dis_arbs_other<br>Show the field ONLY if:<br>[dis_arbs_type] = '7'	If other ARBs, specify which	text, Required
268	dis_abrs_dose<br>Show the field ONLY if:<br>[dis_arbs] = '1'	ARBs TOTAL daily dose (mg)<br>*If unknown use 999.*	text (number, Min: 0, Max: 999), Required
269	dis_arni<br>Show the field ONLY if:<br>[hospitalization] = '1' and [inhosp_out_mortality] = '0'	Angiotensin receptor neprilysin inhibitor (ARNI)<br>*Sacubitril/valsartan (Entresto)*	yesno, Required<br>1 Yes<br>0 No
270	dis_arni_dose<br>Show the field ONLY if:<br>[dis_arni] = '1'	Angiotensin receptor neprilysin inhibitor (ARNI) total daily dose (mg)<br>*Sacubitril/valsartan (Entresto). If unknown use 999.*	text (number, Min: 0, Max: 999), Required
271	dis_statins<br>Show the field ONLY if:<br>[hospitalization] = '1' and [inhosp_out_mortality] = '0'	Statins	yesno, Required<br>1 Yes<br>0 No
272	dis_statin_type<br>Show the field ONLY if:<br>[dis_statins] = '1'	Statin type	radio, Required<br>1 Atorvastatin<br>2 Rosuvastatin<br>3 Simvastatin<br>4 Other
273	dis_statin_other<br>Show the field ONLY if:<br>[dis_statin_type] = '4'	If other statin, specify which	text, Required
274	dis_statin_dose<br>Show the field ONLY if:<br>[dis_statins] = '1'	Statin TOTAL daily dose (mg)<br>*If unknown use 999.*	text (number, Min: 0, Max: 999), Required
275	dis_beta_block<br>Show the field ONLY if:<br>[hospitalization] = '1' and [inhosp_out_mortality] = '0'	Beta Blocker	yesno, Required<br>1 Yes<br>0 No
276	dis_beta_block_type<br>Show the field ONLY if:	Beta Blocker type	radio, Required<br>1 Bisoprolol

	[dis_beta_block] = '1'		2 Atenolol<br>3 Metoprolol<br>4 Carvedilol<br>5 Other
277	dis_beta_block_other<br>Show the field ONLY if:<br>[dis_beta_block_type] = '5'	If other Beta blocker, specify which	text, Required
278	dis_beta_block_dose<br>Show the field ONLY if:<br>[dis_beta_block] = '1'	Beta Blocker TOTAL daily dose (mg)<br>*If unknown use 999.*	text (number, Min: 0, Max: 999), Required
279	dis_ccb<br>Show the field ONLY if:<br>[hospitalization] = '1' and [inhosp_out_mortality] = '0'	Calcium channel blocker	yesno, Required<br>1 Yes<br>0 No
280	dis_ccb_type<br>Show the field ONLY if:<br>[dis_ccb] = '1'	Calcium channel blockers type	radio, Required<br>1 Amlodipine<br>2 Diltiazem<br>3 Nicardipine<br>4 Nifedipine<br>5 Verapamil<br>6 Other
281	dis_ccb_other<br>Show the field ONLY if:<br>[dis_ccb_type] = '6'	If other calcium channel blocker, specify which	text, Required
282	dis_ccb_dose<br>Show the field ONLY if:<br>[dis_ccb] = '1'	Calcium channel blocker TOTAL daily dose (mg)<br>*If unknown use 999.*	text (number, Min: 0, Max: 999), Required
283	dis_loop_diuretics<br>Show the field ONLY if:<br>[hospitalization] = '1' and [inhosp_out_mortality] = '0'	Loop diuretic<br>*Example: Furosemide, Torsemide, etc.*	yesno, Required<br>1 Yes<br>0 No
284	dis_loop_diuretic_dose<br>Show the field ONLY if:<br>[dis_loop_diuretics] = '1'	Loop diuretic TOTAL daily dose (mg)<br>*If unknown use 999.*	text (number, Min: 0, Max: 999), Required
285	dis_mras<br>Show the field ONLY if:<br>[hospitalization] = '1' and [inhosp_out_mortality] = '0'	Mineralocorticoid receptor antagonists (MRAs)<br>*Spironolactone or eplerenone*	yesno, Required<br>1 Yes<br>0 No
286	dis_mras_type<br>Show the field ONLY if:<br>[dis_mras] = '1'	Mineralocorticoid receptor antagonists type	radio, Required<br>1 Spironolactone<br>2 Eplerenone
287	dis_mras_dose<br>Show the field ONLY if:<br>[dis_mras] = '1'	Mineralocorticoid receptor antagonists (MRAs) TOTAL daily dose (mg)<br>*If unknown use 999.*	text (number, Min: 0, Max: 999), Required
288	dis_ppis<br>Show the field ONLY if:<br>[hospitalization] = '1' and [inhosp_out_mortality] = '0'	Proton-pump inhibitors	yesno, Required<br>1 Yes<br>0 No
289	dis_oral_hypo_glc<br>Show the field ONLY if:<br>[hospitalization] = '1' and [inhosp_out_mortality] = '0'	Oral hypoglycemic agents	yesno, Required<br>1 Yes<br>0 No
290	dis_insulin<br>Show the field ONLY if:	Insulin	yesno, Required<br>1 Yes

	Show the field ONLY if:<br>[hospitalization] = '1' and [inhosp_out_mortality] = '0'		0 No
291	dis_anticoagulant<br>Show the field ONLY if:<br>[hospitalization] = '1' and [inhosp_out_mortality] = '0'	Was the patient treated with any anticoagulant therapy?<br>*Example: acenocoumarol, enoxaparin, rivaroxaban, etc.*	yesno, Required<br>1 Yes<br>0 No
292	dis_anticoagulant_type<br>Show the field ONLY if:<br>[dis_anticoagulant] = '1'	Which type of Anticoagulant treatment?<br>**Example: Rivaroxaban, dabigatran, apixaban, or edoxaban.*	radio, Required<br>1 Vitamin K antagonists (acenocoumarol)<br>2 Directly acting oral anticoagulants*<br>3 Low-molecular-weight heparin (Enoxaparin, et
293	dis_anticoagulant_type_2nd<br>Show the field ONLY if:<br>[dis_anticoagulant] = '1'	If the patient was treated with a second anticoagulant treatment, specify which<br>**Example: Rivaroxaban, dabigatran, apixaban, or edoxaban.*	radio<br>1 Vitamin K antagonists (acenocoumarol)<br>2 Directly acting oral anticoagulants*<br>3 Low-molecular-weight heparin (Enoxaparin, et
294	dis_anticoagulant_why<br>Show the field ONLY if:<br>[dis_anticoagulant] = '1'	Clinical indication for anticoagulant treatment	radio, Required<br>1 Prophylaxis<br>2 Atrial fibrillation<br>3 Venous thromboembolism<br>4 Pulmonary embolism<br>5 Arterial embolism<br>6 Stroke<br>7 Cardiac intraventricular thrombus<br>8 Other
295	dis_anticoagulant_other<br>Show the field ONLY if:<br>[dis_anticoagulant_why] = '8'	If other indication for anticoagulant treatment, specify which	text
296	dis_anticoagulant_why_2<br>Show the field ONLY if:<br>[dis_anticoagulant] = '1'	If there was a second clinical indication for anticoagulant treatment, specify which	radio<br>1 Prophylaxis<br>2 Atrial fibrillation<br>3 Venous thromboembolism<br>4 Pulmonary embolism<br>5 Arterial embolism<br>6 Stroke<br>7 Cardiac intraventricular thrombus
297	dis_lwmh_tx_type<br>Show the field ONLY if:<br>[dis_anticoagulant_type] = '3' or [dis_anticoagulant_type_2nd] = '3'	Which Low-molecular-weight heparin was used?	radio, Required<br>1 Enoxaparin<br>2 Dalteparin<br>3 Bemiparin<br>4 Tinzaparin<br>5 Fondaparinux<br>6 Nadroparin<br>7 Certoparin<br>8 Other
298	dis_anticoag_other<br>Show the field ONLY if:<br>[dis_lwmh_tx_type] = '8'	If other Low-molecular-weight heparin, specify which	text, Required
299	dis_lmwh_dose<br>Show the field ONLY if:<br>[dis_anticoagulant_type] = '3' or [dis_anticoagulant_type_2nd] = '3'	Specify the highest dose of Low-molecularweight heparin (only numbers) that was used.<br>*Example: 40 mg or 4000 UI (only numbers).*	text (number, Min: 0, Max: 10000000)
300	dis_lmwh_unit	Which was the Low-molecular-weight heparin [illegible]	radio, Required

300	dis_lmwh_unit<br><br>Show the field ONLY if:<br>[dis_anticoagulant_type] = '3' or [dis_anticoagulant_type_2nd] = '3'	Which was the Low-molecular-weight heparin Unit?	radio, Required<br>1 mg<br>2 UI
301	dis_lmwh_frequency<br><br>Show the field ONLY if:<br>[dis_anticoagulant_type] = '3' or [dis_anticoagulant_type_2nd] = '3'	Which was the frequency of administration of the Low-molecular-weight heparin?	radio<br>1 Each 24 hours<br>2 Each 12 hours
302	dis_noac_type<br><br>Show the field ONLY if:<br>[dis_anticoagulant_type] = '2' or [dis_anticoagulant_type_2nd] = '2'	If Directly acting oral anticoagulants were used, specify which	radio, Required<br>1 Rivaroxaban (Xarelto)<br>2 Dabigatran (Pradaxa)<br>3 Apixaban (Eliquis)<br>4 Edoxaban (Lixiana)
303	dis_noac_dose<br><br>Show the field ONLY if:<br>[dis_anticoagulant_type] = '2' or [dis_anticoagulant_type_2nd] = '2'	Specify the Directly acting oral anticoagulants TOTAL Daily dose (mg)<br>*If unknown use 999.*	text (number, Min: 0, Max: 999), Required
304	dis_nsaids<br><br>Show the field ONLY if:<br>[hospitalization] = '1' and [inhosp_out_mortality] = '0'	Nonsteroidal anti-inflammatory drugs (NSAIDs)	yesno, Required<br>1 Yes<br>0 No
305	dis_nsaids_type<br><br>Show the field ONLY if:<br>[dis_nsaids] ='1'	NSAIDs type	radio, Required<br>1 Ibuprofen<br>2 Diclofenac<br>3 Naproxen<br>4 Dexketoprofen or ketoprofen<br>5 Celecoxib<br>6 Other
306	dis_nsaids_dose<br><br>Show the field ONLY if:<br>[dis_nsaids] ='1'	NSAIDs total daily dose (mg)<br>*If unknown use 9999.*	text (number, Min: 0, Max: 9999), Required
307	dis_cortis<br><br>Show the field ONLY if:<br>[hospitalization] = '1' and [inhosp_out_mortality] = '0'	Corticosteroids therapy<br>*Example: Methylprednisolone, prednisone, dexamethasone, etc.*	yesno, Required<br>1 Yes<br>0 No
308	dis_cortis_type<br><br>Show the field ONLY if:<br>[dis_cortis] ='1'	Specify which type of Corticosteroids	radio, Required<br>1 Methylprednisolone<br>2 Dexamethasone<br>3 Prednisone<br>4 Prednisolone<br>5 Hydrocortisone<br>6 Other
309	dis_cortis_other<br><br>Show the field ONLY if:<br>[dis_cortis_type] = '6'	If other corticosteroids, specify which:	text, Required
310	dis_cortis_dose<br><br>Show the field ONLY if:<br>[dis_cortis] ='1'	Specify the dose of corticosteroids (mg)?<br>*Example: 20 mg (only numbers).*	text
311	dis_another_tx<br><br>Show the field ONLY if:<br>[hospitalization] = '1' and [inhosp_out_mortality] = '0'	If there was any other relevant medication, specify which<br>*Use a comma to separate.*	notes

312	discharge_medications_outcomes_complete	Section Header: *Form Status*<br>Complete?	dropdown<br>0 Incomplete<br>1 Unverified<br>2 Complete
Instrument: **@1-year Outcomes & Medications** (year_outcomes_medications)			Collaps
313	fu1y_done	Section Header: *@1-year FOLLOW-UP OUTCOMES*<br>1-year follow-up was performed	yesno, Required<br>1 Yes<br>0 No
314	fu1y_date<br>Show the field ONLY if:<br>[fu1y_done] ='1'	Date of Follow-up	text (date_dmy, Min: 2020-02-01), Required
315	fu1y_new_swab<br>Show the field ONLY if:<br>[fu1y_done] ='1'	Did the patient has undergone a new Coronavirus swab?<br>*Between baseline and follow-up.*	yesno, Required<br>1 Yes<br>0 No
316	fu1y_new_swab_date<br>Show the field ONLY if:<br>[fu1y_done] ='1' and [fu1y_new_swab] = '1'	If the patient performed a new Coronavirus swab, indicate the date	text (date_dmy, Min: 2020-02-01), Required
317	fu1y_new_swab_result<br>Show the field ONLY if:<br>[fu1y_done] ='1' and [fu1y_new_swab] = '1'	If the patient has undergone a new swab, specify the result.	radio, Required<br>0 Negative<br>1 Positive
318	fu1y_antibody_test<br>Show the field ONLY if:<br>[fu1y_done] ='1'	Did the patient has undergone a Coronavirus antibody test?<br>*Between baseline and follow-up.*	yesno, Required<br>1 Yes<br>0 No
319	fu1y_antibody_date<br>Show the field ONLY if:<br>[fu1y_done] ='1' and [fu1y_antibody_test] = '1'	If the patient has undergone a Coronavirus antibody test, specify the date	text (date_dmy, Min: 2020-02-01), Required
320	fu1y_antibody_igg<br>Show the field ONLY if:<br>[fu1y_done] ='1' and [fu1y_antibody_test] = '1'	If the patient has undergone an antibody test, specify the result of IgG.	radio<br>0 Negative<br>1 Positive
321	fu1y_antibody_igm<br>Show the field ONLY if:<br>[fu1y_done] ='1' and [fu1y_antibody_test] = '1'	If the patient has undergone an antibody test, specify the result of IgM.	radio<br>0 Negative<br>1 Positive
322	fu1y_antibody_iga<br>Show the field ONLY if:<br>[fu1y_done] ='1' and [fu1y_antibody_test] = '1'	If the patient has undergone an antibody test, specify the result of IgA.	radio<br>0 Negative<br>1 Positive
323	fu1y_vaccine<br>Show the field ONLY if:<br>[fu1y_done] ='1' and [coordinating_center] = '1'	Does the patient was vaccinated?	yesno, Required<br>1 Yes<br>0 No
324	fu1y_vaccine_date<br>Show the field ONLY if:<br>[fu1y_vaccine]='1'	Date of the first dose	text (date_dmy, Min: 2020-02-01), Required
325	fu1y_vaccine_type<br>Show the field ONLY if:<br>[fu1y_vaccine]='1'	Which vaccine was used?	radio, Required<br>1 Pfizer<br>2 Moderna<br>3 AstraZeneca<br>4 Janssen<br>5 Other

326	fu1y_vaccione_type_other<br>Show the field ONLY if:<br>[fu1y_vaccine_type]='5'	If other, specify	text, Required
327	fu1y_vaccine_doses<br>Show the field ONLY if:<br>[fu1y_vaccine]='1'	Is the vaccination regimen complete?	radio, Required<br>1 One dose (only for 2 doses vaccines: Pfizer, Moderna or AstraZeneca)<br>2 Full vaccinated
328	fu1y_rehosp<br>Show the field ONLY if:<br>[fu1y_done] ='1'	Section Header: *RE-HOSPITALIZATION*<br>RE-HOSPITALIZATION	yesno, Required<br>1 Yes<br>0 No
329	fu1y_hosp_cause<br>Show the field ONLY if:<br>[fu1y_rehosp] = '1'	Did the re-hospitalization was related to a COVID-19?<br>*Consider any complication or sequelae related to COVID-19.*	yesno, Required<br>1 Yes<br>0 No
330	fu1y_rehosp_specify<br>Show the field ONLY if:<br>[fu1y_rehosp] = '1'	Specify the diagnosis (diagnosis/specialty) of the re-hospitalization?<br>*Use a comma to separate. Example: stroke,neurology*	notes
331	fu1y_rehosp_quantity<br>Show the field ONLY if:<br>[fu1y_rehosp] ='1'	If the patient had more than one hospitalization, specify the number.	text (number, Min: 2, Max: 100)
332	fu1y_rehosp_icu<br>Show the field ONLY if:<br>[fu1y_done] ='1' and [fu1y_rehosp] = '1'	Did the patient require admission in an intensive care unit?<br>*In any re-hospitalization.*	yesno, Required<br>1 Yes<br>0 No
333	fu1y_rehosp_imv<br>Show the field ONLY if:<br>[fu1y_rehosp_icu] = '1'	Mechanical ventilation<br>*In any re-hospitalization.*	yesno, Required<br>1 Yes<br>0 No
334	fu1y_out_mortality<br>Show the field ONLY if:<br>[fu1y_done] ='1'	Section Header: *@1-YEAR OUTCOMES*<br>-- 1-YEAR PATIENT MORTALITY --<br>(PRIMARY ENDPOINT)	yesno, Required<br>1 Yes<br>0 No
335	fu1y_out_mortality_date<br>Show the field ONLY if:<br>[fu1y_out_mortality] = '1'	Day of death	text (date_dmy, Min: 2020-02-01), Required
336	fu1y_out_cv_death<br>Show the field ONLY if:<br>[fu1y_out_mortality] = '1'	Suspected cardiovascular death	radio<br>1 Death caused by acute myocardial infarction<br>2 Death caused by sudden cardiac, including unwitnessed<br>3 Death resulting from heart failure<br>4 Death caused by stroke<br>5 Death caused by cardiovascular procedures<br>6 Death resulting from cardiovascular hemorrha<br>7 Death resulting from other cardiovascular caus
337	fu1y_out_noncv_death<br>Show the field ONLY if:<br>[fu1y_out_mortality] = '1'	Suspected Non-cardiovascular death	radio<br>1 Death resulting from malignancy<br>2 Death resulting from pulmonary causes<br>3 Death caused by infection (includes sepsis)<br>4 Death resulting from gastrointestinal causes<br>5 Death resulting from accident/trauma<br>6 Death caused by other noncardiovascular orga failure<br>7 Death resulting from other noncardiovascular cause<br>8 Undetermined cause of death

			[illegible] Undetermined cause of death
338	fu1y_death_report<br>Show the field ONLY if:<br>[fu1y_out_mortality] = '1'	Anonymize and upload the discharge (mortality) report. Document needed for death adjudication.	file
339	fu1y_out_acs<br>Show the field ONLY if:<br>[fu1y_done] ='1'	1-YEAR FOLLOW-UP ACS<br>(Acute Coronary Syndrome)	yesno, Required<br>1 Yes<br>0 No
340	fu1y_out_acs_type<br>Show the field ONLY if:<br>[fu1y_out_acs] = '1'	Which type of Acute Coronary Syndrome?	radio, Required<br>1 Unstable angina<br>2 NSTEMI<br>3 STEMI
341	fu1y_out_acs_date<br>Show the field ONLY if:<br>[fu1y_out_acs] = '1'	Date of the ACS?	text (date_dmy, Min: 2020-02-01), Required
342	fu1y_out_revasc<br>Show the field ONLY if:<br>[fu1y_done] ='1'	1-YEAR FOLLOW-UP CORONARY REVASCULARIZATION	yesno, Required<br>1 Yes<br>0 No
343	fu1y_out_revasc_type<br>Show the field ONLY if:<br>[fu1y_out_revasc] = '1'	Which type of coronary Revascularization?	radio, Required<br>1 PCI<br>2 CABG
344	fu1y_out_revasc_date<br>Show the field ONLY if:<br>[fu1y_out_revasc] = '1'	Date of revascularization	text (date_dmy, Min: 2020-02-01), Required
345	fu1y_out_tlr<br>Show the field ONLY if:<br>[fu1y_out_revasc] = '1'	If the patient had a revascularization, it was a target lesion revascularization?<br>*Target lesion revascularization, refers to a stent implanted during the index hospitalization*	yesno, Required<br>1 Yes<br>0 No
346	fu1y_out_stent_thrombo<br>Show the field ONLY if:<br>[fu1y_out_revasc] = '1'	If the patient had revascularization, it was related to a stent thrombosis?	yesno, Required<br>1 Yes<br>0 No
347	fu1y_stroke<br>Show the field ONLY if:<br>[fu1y_done] ='1'	1-YEAR FOLLOW-UP STROKE/TIA<br>*TIA: transient ischemic attack*	yesno, Required<br>1 Yes<br>0 No
348	fu1y_out_stroke_type<br>Show the field ONLY if:<br>[fu1y_stroke] = '1'	Which type of Stroke/transient ischemic attack the patient had?	radio, Required<br>1 Ischemic stroke<br>2 Transient ischemic attack<br>3 Hemorrhagic stroke
349	fu1y_out_stroke_date<br>Show the field ONLY if:<br>[fu1y_stroke] = '1'	Date of the Stroke/transient ischemic attack	text (date_dmy, Min: 2020-02-01), Required
350	fu1y_out_hf<br>Show the field ONLY if:<br>[fu1y_done] ='1'	1-YEAR FOLLOW-UP HEART FAILURE HOSPITALIZATION	yesno, Required<br>1 Yes<br>0 No
351	fu1y_out_hf_nyha<br>Show the field ONLY if:<br>[fu1y_out_hf] = '1'	Which class of New York Heart Association did the patient presented?<br>*NYHA: New York Heart Association.*	radio, Required<br>1 NYHA I<br>2 NYHA II<br>3 NYHA III<br>4 NYHA IV
352	fu1y_out_ahf_date<br>Show the field ONLY if:<br>[fu1y_out_hf] = '1'	Date of Heart failure hospitalization admission.	text (date_dmy, Min: 2020-02-01), Required

353	fu1y_out_vte<br>Show the field ONLY if:<br>[fu1y_done] ='1'	1-YEAR FOLLOW-UP VENOUS/ARTERIAL THROMBOEMBOLISM	yesno, Required<br>1 Yes<br>0 No
354	fu1y_out_vte_type<br>Show the field ONLY if:<br>[fu1y_out_vte] = '1'	Which type of venous/arterial thromboembolism?	radio, Required<br>1 Deep vein thrombosis<br>2 Arterial embolism
355	fu1y_out_vte_date<br>Show the field ONLY if:<br>[fu1y_out_vte] = '1'	Date of Venous/arterial thromboembolism	text (date_dmy, Min: 2020-02-01), Required
356	fu1_out_vte_describe<br>Show the field ONLY if:<br>[fu1y_out_vte] = '1'	If the patient presented a venous/arterial thromboembolism, describe the location and if invasive treatment was needed.<br>*Use a comma to separate the diagnosis, location, and treatment.*	notes
357	fu1y_out_pe<br>Show the field ONLY if:<br>[fu1y_done] ='1'	1-YEAR FOLLOW-UP PULMONARY EMBOLISM	yesno, Required<br>1 Yes<br>0 No
358	fu1y_out_pe_laterality<br>Show the field ONLY if:<br>[fu1y_out_pe] = '1'	If the patient had a Pulmonary embolism, specify the laterality	radio, Required<br>1 Right<br>2 left<br>3 Both
359	fu1y_out_pe_vessel<br>Show the field ONLY if:<br>[fu1y_out_pe] = '1'	If the patient had a Pulmonary embolism, specify which type	radio, Required<br>1 Major vessel (Main artery, Lobar artery, Segmental)<br>2 Minor vessel (Sub-segmental)
360	nhosp_out_pe_date<br>Show the field ONLY if:<br>[fu1y_out_pe] = '1'	Date of Pulmonary embolism	text (date_dmy, Min: 2020-02-01), Required
361	fu1y_out_bleeding<br>Show the field ONLY if:<br>[fu1y_done] ='1'	1-YEAR FOLLOW-UP MAJOR BLEEDING	yesno, Required<br>1 Yes<br>0 No
362	fu1y_out_bleeding_type<br>Show the field ONLY if:<br>[fu1y_out_bleeding] = '1'	If the patient presented a major bleeding, specify which type?	radio, Required<br>1 Bleeding with an Hb drop between 3-5 g/dL.<br>2 Bleeding requiring transfusion<br>3 Bleeding requiring surgical intervention<br>4 Non-fatal intracranial hemorrhage<br>5 Fatal bleeding without autopsy/imaging confirmation<br>6 Fatal bleeding with autopsy/imaging confirmation
363	fu1y_out_bleeding_date<br>Show the field ONLY if:<br>[fu1y_out_bleeding] = '1'	Date of Major bleeding	text (date_dmy, Min: 2020-02-01), Required
364	fu1y_out_bleeding_site<br>Show the field ONLY if:<br>[fu1y_out_bleeding] = '1'	Specify the site of origin if the major bleeding:	radio, Required<br>1 Intracraneal or intraspinal<br>2 Pulmonary (hemoptysis, endotracheal tube, bronchoscopy)<br>3 Gastrointestinal (hematemesis, melena, uppe endoscopy or colonoscopy)<br>4 Genito-urinary (urine, urinary catheter, cystoscopy)<br>5 Nose (Epistaxis)<br>6 Soft tissue hematoma<br>7 Solid organ hematoma<br>8 Ocular

			9 Intra-abdominal bleeding<br>10 Retroperitoneal bleeding<br>11 Other
365	fu1y_out_bleeding_other<br><br>Show the field ONLY if:<br>[fu1y_out_bleeding_site]=11	If other, describe the site of origin:	text, Required
366	fu1y_bleeding_descrip<br><br>Show the field ONLY if:<br>[fu1y_out_bleeding] = '1'	Provide a very small description of the bleeding event	notes, Required
367	fu1y_out_transfusion<br><br>Show the field ONLY if:<br>[fu1y_done] ='1'	1-YEAR FOLLOW-UP RED BLOOD CELL TRANSFUSION	yesno, Required<br>1 Yes<br>0 No
368	fu1y_out_arrhyth<br><br>Show the field ONLY if:<br>[fu1y_done] ='1'	1-YEAR FOLLOW-UP SERIOUS ARRHYTHMIAS EVENTS	yesno, Required<br>1 Yes<br>0 No
369	fu1y_out_arrhyth_type<br><br>Show the field ONLY if:<br>[fu1y_out_arrhyth] = '1'	If the patient presented serious arrhythmias events, specify which	radio, Required<br>1 Bradicardya requiring pacemaker<br>2 New onset of atrial fibrillation or flutter<br>3 Any episode of Ventricular tachycardia
370	fu1y_out_arrhyth_date<br><br>Show the field ONLY if:<br>[fu1y_out_arrhyth] = '1'	Date of presentation of Cardiac arrhythmia	text (date_dmy, Min: 2020-02-01), Required
371	fu1y_lvef<br><br>Show the field ONLY if:<br>[fu1y_done] = '1'	Left-Ventricular Ejection Fraction (%) (LVEF)<br>*Assessed by any method. If unknown use 999.*	text (number, Min: 0, Max: 999), Required
372	fu1y_other_events<br><br>Show the field ONLY if:<br>[fu1y_done] ='1'	If there was another relevant event, specify which.<br>*Use a comma to separate the complications. Example: urgent surgery, etc.*	notes
373	fu1y_aspirin<br><br>Show the field ONLY if:<br>[fu1y_done] ='1' and [fu1y_out_mortality] = '0'	Section Header: *@1-year FOLLOW-UP MEDICATIONS*<br>Aspirin	yesno, Required<br>1 Yes<br>0 No
374	fu1y_p2y12<br><br>Show the field ONLY if:<br>[fu1y_done] ='1' and [fu1y_out_mortality] = '0'	P2Y12 inhibitor	yesno, Required<br>1 Yes<br>0 No
375	fu1y_p2y12_type<br><br>Show the field ONLY if:<br>[fu1y_p2y12] = '1'	Which P2Y12 inhibitor	radio, Required<br>1 Clopidogrel<br>2 Prasugrel<br>3 Ticagrelor
376	fu1y_ace_inh<br><br>Show the field ONLY if:<br>[fu1y_done] ='1' and [fu1y_out_mortality] = '0'	ACE inhibitor<br>*Angiotensin-converting-enzyme inhibitors (ACE inhibitors)*	yesno, Required<br>1 Yes<br>0 No
377	fu1y_ace_type<br><br>Show the field ONLY if:<br>[fu1y_ace_inh] = '1'	ACE inhibitor type	radio, Required<br>1 Lisinopril<br>2 Enalapril<br>3 Perindopril<br>4 Other
378	fu1y_ace_inh_other<br><br>Show the field ONLY if:<br>[fu1y_ace_type] = '4'	If other ACE inhibitor specify	text, Required
[illegible]	[illegible]	[illegible]	[illegible]

379	fu1y_ace_dose<br>Show the field ONLY if:<br>[fu1y_ace_inh] = '1'	ACE inhibitor TOTAL daily dose (mg)<br>*If unknown use 999.*	text (number, Min: 1, Max: 999), Required
380	fu1y_arbs<br>Show the field ONLY if:<br>[fu1y_done] ='1' and [fu1y_ou t_mortality] = '0'	ARBs<br>*Angiotensin II receptor blockers*	yesno, Required<br>1 Yes<br>0 No
381	fu1y_arbs_type<br>Show the field ONLY if:<br>[fu1y_arbs] = '1'	ARBs type	radio, Required<br>1 Losartan<br>2 Candesartan<br>3 Irbesartan<br>4 Telmisartan<br>5 Valsartan<br>6 Olmesartan<br>7 Other
382	fu1y_arbs_other<br>Show the field ONLY if:<br>[fu1y_arbs_type] = '7'	If other ARBs specify	text, Required
383	fu1y_arbs_dose<br>Show the field ONLY if:<br>[fu1y_arbs] = '1'	ARBs TOTAL daily dose (mg)<br>*If unknown use 999.*	text (number, Min: 0, Max: 999), Required
384	fu1y_arni<br>Show the field ONLY if:<br>[fu1y_done] ='1' and [fu1y_ou t_mortality] = '0'	Angiotensin receptor neprilysin inhibitor (ARNI)<br>*Sacubitril/valsartan (Entresto)*	yesno, Required<br>1 Yes<br>0 No
385	fu1y_arni_dose<br>Show the field ONLY if:<br>[fu1y_arni] = '1'	Angiotensin receptor neprilysin inhibitor (ARNI) TOTAL daily dose (mg)<br>*Sacubitril/valsartan (Entresto). If unknown use 999.*	text (number, Min: 0, Max: 999), Required
386	fu1y_statins<br>Show the field ONLY if:<br>[fu1y_done] ='1' and [fu1y_ou t_mortality] = '0'	Statins	yesno, Required<br>1 Yes<br>0 No
387	fu1y_statin_type<br>Show the field ONLY if:<br>[fu1y_statins] = '1'	Statin type	radio, Required<br>1 Atorvastatin<br>2 Rosuvastatin<br>3 Other
388	fu1y_statin_other<br>Show the field ONLY if:<br>[fu1y_statin_type] = '3'	If other statin specify	text
389	fu1y_statin_dose<br>Show the field ONLY if:<br>[fu1y_statins] = '1'	Statin TOTAL daily dose (mg)<br>*If unknown use 999.*	text (number, Min: 0, Max: 999)
390	fu1y_betablocker<br>Show the field ONLY if:<br>[fu1y_done] ='1' and [fu1y_ou t_mortality] = '0'	Beta blocker	yesno, Required<br>1 Yes<br>0 No
391	fu1y_beta_block_type<br>Show the field ONLY if:<br>[fu1y_betablocker] = '1'	Beta Blocker type	radio, Required<br>1 Bisoprolol<br>2 Atenolol<br>3 Metoprolol<br>4 Carvedilol<br>5 Other
392	fu1y_beta_block_other	If other beta blocker specify	text, Required

	Show the field ONLY if: [fu1y_beta_block_type] = '5'		
393	fu1y_beta_block_dose<br>Show the field ONLY if: [fu1y_betablocker] = '1'	Beta Blocker TOTAL daily dose (mg)<br>*If unknown use 999.*	text (number, Min: 0, Max: 999), Required
394	fu1y_ccb<br>Show the field ONLY if: [fu1y_done] ='1' and [fu1y_out_mortality] = '0'	Calcium channel blocker	yesno, Required<br>1 Yes<br>0 No
395	fu1y_ccb_type<br>Show the field ONLY if: [fu1y_ccb] = '1'	Calcium channel blockers type	radio, Required<br>1 Amlodipine<br>2 Diltiazem<br>3 Nicardipine<br>4 Nifedipine<br>5 Verapamil<br>6 Other
396	fu1y_ccb_other<br>Show the field ONLY if: [fu1y_ccb_type] = '6'	If other calcium channel blocker specify	text, Required
397	fu1y_ccb_dose<br>Show the field ONLY if: [fu1y_ccb] = '1'	Calcium channel blocker TOTAL daily dose (mg)<br>*If unknown use 999.*	text (number, Min: 0, Max: 999), Required
398	fu1y_loop_diuretics<br>Show the field ONLY if: [fu1y_done] ='1' and [fu1y_out_mortality] = '0'	Loop diuretic<br>*Example: Furosemide, Torsemide, etc.*	yesno, Required<br>1 Yes<br>0 No
399	fu1y_loop_diuretic_dose_2<br>Show the field ONLY if: [fu1y_loop_diuretics] = '1'	TOTAL daily dose of loop diuretic (mg)<br>*If unknown use 999.*	text (number, Min: 0, Max: 999)
400	fu1y_mras<br>Show the field ONLY if: [fu1y_done] ='1' and [fu1y_out_mortality] = '0'	Mineralocorticoid receptor antagonists (MRAs)<br>*Spironolactone or eplerenone*	yesno, Required<br>1 Yes<br>0 No
401	fu1y_mras_type<br>Show the field ONLY if: [fu1y_mras] = '1'	Mineralocorticoid receptor antagonists type	radio, Required<br>1 Spironolactone<br>2 Eplerenone
402	fu1y_mras_dose<br>Show the field ONLY if: [fu1y_mras] = '1'	Mineralocorticoid receptor antagonists (MRAs) TOTAL daily dose (mg)<br>*If unknown use 999.*	text (number, Min: 0, Max: 999)
403	fu1y_oralhypoglc<br>Show the field ONLY if: [fu1y_done] ='1' and [fu1y_out_mortality] = '0'	Oral hypoglycemic agents	yesno, Required<br>1 Yes<br>0 No
404	fu1y_insulin<br>Show the field ONLY if: [fu1y_done] ='1' and [fu1y_out_mortality] = '0'	Insulin	yesno, Required<br>1 Yes<br>0 No
405	fu1y_anticoagulant<br>Show the field ONLY if: [fu1y_done] ='1' and [fu1y_out_mortality] = '0'	Anticoagulant treatment	yesno, Required<br>1 Yes<br>0 No
406	fu1y_anticoagulant_type<br>Show the field ONLY if: [fu1y_anticoagulant] = '1'	Anticoagulant treatment<br>**Example: Rivaroxaban, dabigatran, apixaban, or edoxaban.*	radio, Required<br>1 Vitamin K antagonists (acenocumarol)<br>2 Directly acting oral anticoagulants*<br>3 Low-molecular-weight heparin (Enoxaparin, et[illegible]

407	fu1y_anticoagulant_type2<br>Show the field ONLY if:<br>[fu1y_anticoagulant] = '1'	If the patient had received a second anticoagulant treatment, specify which<br>**Example: Rivaroxaban, dabigatran, apixaban, or edoxaban.*	radio<br>1 Vitamin K antagonists (acenocumarol)<br>2 Directly acting oral anticoagulants*<br>3 Low-molecular-weight heparin (Enoxaparin, et[illegible]
408	fu1y_anticoagulant_why<br>Show the field ONLY if:<br>[fu1y_anticoagulant] = '1'	Clinical indication for anticoagulation	radio, Required<br>1 Atrial fibrillation<br>2 Venous thromboembolism<br>3 Pulmonary embolism<br>4 Arterial embolism<br>5 Stroke<br>6 Cardiac intraventricular thrombus<br>7 Prophylaxis
409	fu1y_anticoagulant_why_2<br>Show the field ONLY if:<br>[fu1y_anticoagulant] = '1'	If there is a second clinical indication for anticoagulation, please detail	radio<br>1 Atrial fibrillation<br>2 Venous thromboembolism<br>3 Pulmonary embolism<br>4 Arterial embolism<br>5 Stroke<br>6 Cardiac intraventricular thrombus<br>7 Prophylaxis
410	fu1y_lwmh_tx_type<br>Show the field ONLY if:<br>[fu1y_anticoagulant_type] = '3' or [fu1y_anticoagulant_type2] = '3'	Please select the used Low-molecular-weight heparin	radio, Required<br>1 Enoxaparin<br>2 Dalteparin<br>3 Bemiparin<br>4 Tinzaparin<br>5 Fondaparinux<br>6 Nadroparin<br>7 Certoparin<br>8 Other
411	fu1y_anticoag_other<br>Show the field ONLY if:<br>[fu1y_lwmh_tx_type] = '8'	If other, please specify the type of Low-molecular-weight heparin:	text, Required
412	fu1y_lmwh_dose<br>Show the field ONLY if:<br>[fu1y_anticoagulant_type] = '3' or [fu1y_anticoagulant_type2] = '3'	Please provide the dose of Low-molecular-weight heparin (only numbers).<br>*Example: 40 mg or 4000 UI (only numbers). If unknown use 999.*	text (number, Min: 0, Max: 10000000), Required
413	fu1y_lmwh_unit<br>Show the field ONLY if:<br>[fu1y_anticoagulant_type] = '3' or [fu1y_anticoagulant_type2] = '3'	Which is the Low-molecular-weight heparin Unit<br>(mg or UI)	radio, Required<br>1 mg<br>2 UI
414	fu1y_lmwh_frequency<br>Show the field ONLY if:<br>[fu1y_anticoagulant_type] = '3' or [fu1y_anticoagulant_type2] = '3'	Which was the frequency of administration of the Low-molecular-weight heparin	radio, Required<br>1 Each 24 hours<br>2 Each 12 hours
415	fu1y_noac_type<br>Show the field ONLY if:<br>[fu1y_anticoagulant_type] = '2' or [fu1y_anticoagulant_type2] = '2'	If Novel oral anticoagulants was used which one:	radio, Required<br>1 Rivaroxaban (Xarelto)<br>2 Dabigatran (Pradaxa)<br>3 Apixaban (Eliquis)<br>4 Edoxaban (Lixiana)

416	fu1y_noac_dose<br><br>Show the field ONLY if:<br>[fu1y_anticoagulant_type] = '2' or [fu1y_anticoagulant_type2] = '2'	Specify the Directly acting oral anticoagulants TOTAL Daily dose (mg)<br>*If unknown use 999.*	text (number, Min: 0, Max: 999)
417	fu1y_cortis<br><br>Show the field ONLY if:<br>[fu1y_done] ='1' and [fu1y_out_mortality] = '0'	Corticosteroids therapy	yesno, Required<br>1 Yes<br>0 No
418	fu1y_cortis_type<br><br>Show the field ONLY if:<br>[fu1y_cortis] ='1'	Specify which type of Corticosteroids	radio, Required<br>1 Methylprednisolone<br>2 Dexamethasone<br>3 Prednisone<br>4 Prednisolone<br>5 Hydrocortisone<br>6 Other
419	fu1y_cortis_other<br><br>Show the field ONLY if:<br>[fu1y_cortis_type] = '6'	If other corticosteroids, please specify:	text, Required
420	fu1y_cortis_dose<br><br>Show the field ONLY if:<br>[fu1y_cortis] ='1'	Specify the dose of corticosteroids (mg)?<br>*Example: 20 mg (only numbers). If unknown use 999.*	text (number, Min: 0, Max: 999), Required
421	fu1y_other_tx<br><br>Show the field ONLY if:<br>[fu1y_done] ='1' and [fu1y_out_mortality] = '0'	If any other relevant therapy, specify which<br>*Use a comma to separate.*	notes
422	year_outcomes_medications_complete	Section Header: *Form Status*<br><br>Complete?	dropdown<br>0 Incomplete<br>1 Unverified<br>2 Complete
