# Supplementary material for: Long-term effects of coronavirus disease 2019 on the cardiovascular system, CV COVID registry: A structured summary of a study protocol
Source: PLoS One. 2021 Jul 29;16(7):e0255263. doi: 10.1371/journal.pone.0255263 (PMC8320971; doi:10.1371/journal.pone.0255263)
Supplement: S1 Data — (PDF) [file pone.0255263.s004.pdf]

Protocol for retrospective data collection / clinical history review studies

Project title: Effect of Coronavirus 2019 disease on the cardiovascular system in patients who have undergone a diagnostic RT-PCR for SARS-CoV-2.

Project version and date (MANDATORY): 1.0, April 6, 2020.

Project code (if any):

Name and affiliation of the principal investigator of our center (PI): Salvatore Brugaletta.

Name and affiliation of collaborating researchers or second PI (only if any): Luis Ortega Paz and

Manel Sabaté.

Promoter (in case there is an external promoter in the center):

Funding entity (if any or if you apply for a call): none.

Study objective (300 words): Patients with coronavirus disease-2019 are at very high risk of adverse cardiovascular events including death from cardiovascular causes. Unfortunately we do not have reliable statistics on the frequency and severity of these complications. The objective of this study is to determine the frequency of serious cardiovascular complications at one-year follow-up in patients who have had a nasopharyngeal swab for real-time reverse transcriptase-polymerase chain reaction (RT-PCR) for SARS-CoV2 between March 2020 and August 2020.

Main objective: To determine the frequency of cardiovascular death at one year in patients who have had a diagnostic RT-PCR for SARS-CoV-2.

Secondary objectives:

• Determine the rate of serious cardiovascular events such as cardiac death, heart attack cardiac arrest, heart failure and malignant cardiac arrhythmias at one year in patients who have undergone a diagnostic RT-PCR for SARS-CoV-2.

• Determine the cardiovascular risk factors associated with poor prognosis in patients who have performed a diagnostic RT-PCR for SARS-CoV-2.

• Determine the use of angiotensin converting enzyme (ACE) inhibitors, angiotensin receptor blockers (ARB) and the non-steroidal analgesics (NSAIDs) and their relationship with severe manifestations of disease.

Methodology: retrospective study to review the medical records of patients who have performed a diagnostic RT-PCR for SARS-CoV-2 in the emergency department or hospitalization area of the Hospital Clínic of Barcelona. Positive patients will be considered cases, and negative patients as controls. To identify the patients to be included, the discharge census from the emergency room and hospitalization units will be reviewed. Study period March to August, 2020. The source document will be the discharge report.

Variables to collect:

Main variable:

• Vital status at one-year follow-up in patients who have undergone a diagnostic RT-PCR for SARS-CoV-2.

Secondary variables:

• Patient characteristics: demographics and comorbidities, history of cardiovascular diseases and medications; reported in the clinical history of their stay in emergency room or hospital admission.

• Presentation of myocardial infarction, stroke, heart failure and cardiac arrhythmias at one-year follow-up in patients who have undergone a diagnostic RT-PCR for SARS-CoV-2.

• History of use of ACE inhibitors, ARB and NSAIDs during the stay in the emergency room or hospital admission in patients who have undergone a diagnostic RT-PCR for SARS-CoV-2.

• Clinical characteristics and evolution during stay in the emergency room or hospital admission in patients who have undergone a diagnostic RT-PCR for SARS-CoV-2.

• Antiviral / antibiotic drugs used in treatment stay in emergencies or hospital admission in patients who have undergone a diagnostic RT-PCR for SARS-CoV-2.

• Results of cardiovascular tests, electrocardiogram, echocardiogram and cardiac catheterization during the stay in the emergency room or hospital admission in patients who have undergone a diagnostic RT-PCR for SARS-CoV-2.

Center where the study will be performed, number of patients to be included and centers where data are obtained (specify if patient data is collected from other centers):

Hospital Clinic of Barcelona. All patients who have undergone a diagnostic RT-PCR for SARS-CoV-2, and were discharged from the emergency department or the hospitalization area, estimated 1000 patients.

Data source (SAP, own database, etc.): SAP.

> Note: If the source is your own database, you must specify its location, access control, risk management and confirm that the European regulation is followed regarding personal data protection.

People who will have access to the identifying data of the patients (name and affiliation of the people on the research team):

• Dr. Manel Sabaté from the Hospital Clínic.

People who will have access to coded patient data (people, with name and surnames, or entities external to the center or research team):

• Dr. Salvatore Brugaletta from the Hospital Clínic de Barcelona.

• Dr. Luis Ortega Paz from IDIBAPS.

Note: We remind you that only those people with their own permission to access the SAP may have access to the identifying data of the patients. In addition, the physician responsible for the patient must provide the coded data to the principal investigator or other team members who do not have access permission own in SAP or in patients ’medical history.

Is it a final degree project, a master's thesis or a doctoral thesis?:

- Yes (specify student name and type of work)
- **No.**

Will informed consent be sought from patients? :

- Yes (in this case, provide the FIP / CI).
- **No, since patients are no longer visited periodically and it is not feasible.**

I, Salvatore Brugaletta, as the principal investigator of the project, promise that that all the research team will process the data as follows:

The processing, communication and transfer of personal data of all participants will comply with EU Regulation 2016/679 of the European Parliament and of the Council of 27 April of 2016 on the protection of individuals with regard to the processing of personal data and the free movement of data and in Organic Law 3/2018 of 5 December on the Protection of Personal Data and Guarantee of digital rights.

The data collected for these studies will be identified only by a code, and it will not include information to identify the participants. Only the physician of the study and its collaborators with the right of access to SAP data, may relate the data collected in the study with the patient’s medical history. The identity of the participants will not be available to anyone other than a medical emergency or legal requirement.

Health authorities, the Ethics Committee of Research and staff authorized by the promoter of the study may have access to personally identifiable information, when necessary to verify data and procedures of the study, but always maintaining confidentiality in accordance with current legislation.

Only the coded data will be transferred to third parties and to other countries, which in no case will it contain information that can identify the participant directly (such as name and surname, initials, address, number of social security, etc.). In the event that such an assignment occurs, it would be for the same purpose of the study described and guaranteeing confidentiality.

I promise to use the data in such a way that it is maintained separate patient identification data from clinic data.

I, Salvatore Brugaletta, as the principal investigator of the project, promise that the study will be carried out in accordance with Law 14/2007 on Biomedical Research and that the Declaration of Helsinki (in its latest version) will be taken into account.

Date: Monday, April 6, 2020.
